# TLR1/2 Specific Small‐Molecule Agonist Suppresses Leukemia Cancer Cell Growth by Stimulating Cytotoxic T Lymphocytes

**DOI:** 10.1002/advs.201802042

**Published:** 2019-03-27

**Authors:** Xiaohong Cen, Gengzhen Zhu, Junjie Yang, Jianjun Yang, Jiayin Guo, Jiabing Jin, Kutty Selva Nandakumar, Wei Yang, Hang Yin, Shuwen Liu, Kui Cheng

**Affiliations:** ^1^ Guangdong Provincial Key Laboratory of New Drug Screening and Guangzhou Key Laboratory of Drug Research for Emerging Virus Prevention and Treatment School of Pharmaceutical Sciences Southern Medical University Guangzhou 510515 China; ^2^ Department of Thoracic Surgery Nanfang Hospital Southern Medical University Guangzhou 510515 China; ^3^ Department of Pathology School of Basic Medical Sciences Department of Pathology Nanfang Hospital, and Guangdong Provincial Key Laboratory of Molecular Oncologic Pathology Southern Medical University Guangzhou 510515 China; ^4^ School of Pharmaceutical Sciences Tsinghua University‐Peking University Joint Center for Life Sciences Tsinghua University Beijing 100082 China

**Keywords:** agonist, CD8^+^ T, leukemia, Toll‐like receptor 2 (TLR2), tumor immunity

## Abstract

Toll‐like receptor 2 (TLR2) expressed on antigen presenting cells evokes a series of critical cytokines, which favor the development of tumor‐specific cytotoxic T lymphocytes (CTLs). Therefore, TLR2 represents an attractive cancer immunotherapeutic target. Here, a synthetic library of 14 000 compounds together with a series of newly developed compounds for NF‐κB activation using HEK‐Blue hTLR2 cells is initially screened. Following further screening in a variety of cells including HEK‐Blue hTLRs reporter cells, murine, and human macrophage cell lines, a potent small molecule agonist 23 (SMU‐Z1) is identified, which specifically activates TLR2 through its association with TLR1, with a EC_50_ of 4.88 ± 0.79 × 10^−9^ m. Toxicology studies, proinflammatory cytokines (e.g., TNF‐α, IL‐1β, IL‐6, and nitric oxide) and target‐protein based biophysical assays demonstrate the pharmacologically relevant characteristics of SMU‐Z1. In addition, SMU‐Z1 promotes murine splenocyte proliferation and upregulates the expression of CD8^+^ T cells, NK cells and DCs, which results in a significant antitumor effect in a murine leukemia model. Finally, the induced tumors in three out of seven mice disappear after administration of SMU‐Z1. Our studies thus identify a novel and potent TLR1/2 small molecule agonist, which displays promising immune adjuvant properties and antitumor immunity.

The progress of mammalian growth and development is always accompanied with their immune system disorders and inflammatory responses.^1^ Toll‐like receptors (TLRs) belong to type I transmembrane protein receptors involved in orchestrating both innate and adaptive immune responses, detecting conserved pathogen‐associated molecular patterns (PAMPs) from microbes as well as in host‐derived danger‐associated molecular patterns (DAMPs).^2^ Until today, several TLR modulators with high efficiency and low toxicity have been discovered that are closely linked to anticancer therapies,^3^ including TLR3 agonist AMP‐516,^4^ TLR5 agonist entolimod (CBLB502),^5^ TLR7 agonist imiquimod,^6^ and TLR9 agonists CpG‐7909^7^ and ISS1018.^8^

As for TLR2, the current TLR2 agonists such as polysaccharide krestin (PSK) extracted from mushrooms is being tested in a clinical phase II trial for the treatment of breast cancer due to its presumed immune potentiating effects.^9^ TLR2 agonists bind to TLR2 on antigen presenting cells (APCs) including dendritic cells (DCs) and macrophages, and initiate antigen presentation, costimulatory molecule expression, and cytokine secretion, thus activate antigen‐specific CD4 T cells and CD8 T cells, which develop cytolytic activity toward tumor cells. PSK has significantly inhibited the breast cancer growth via stimulation of CD8 T cells and natural killer (NK) cells. Other TLR2 agonists or their modified forms, lipopeptides also have been reported to be useful for the cancer treatment,^10^, ^11^, ^12^, ^13^, ^14^, ^15^, ^16^ which further suggests the potential of TLR2 agonist as effective enhancers for cancer immunotherapies.^17^ However, the current known TLR2 agonists, lipopeptides or their derivatives, are having limitations because of the difficulties in synthesis, susceptibility to hydrolysis, as well as in eliciting inflammatory cytokine storm. Therefore, there is a need to develop novel TLR2 agonists with higher stability in vivo, so that they are more suitable for cancer immunotherapy or for their use as immunoadjuvants. Compared to the existing TLR2 agonists, lipopeptide activators or polysaccharides, small molecule agents will have the merits of not easily prone to hydrolysis, low cost of production, high productivity yields, and increased tissue permeability but at the same time without compromising their immunogenic properties. The small molecule activators of TLR have not been studied extensively due to the following reasons: i) TLR needs hetero‐ or homodimerization to initiate the immune responses, and the large protein–protein interfaces make the investigations of protein–protein interactions (PPI) a very challenging task.^18^ ii) There are at least 10 human homologous of TLRs present in murine macrophages, all sharing a ligand‐binding domain with a double‐horseshoe shape and hence it is difficult to search for specific compounds, especially for TLR2, which can heterodimerizes with TLR1 or TLR6. In order to address these issues, as well as to continue our research interests in finding TLR2 modulators,^19^, ^20^, ^21^ we screened a synthetic library of 14 000 compounds and a series of newly developed compounds for NF‐κB activation using HEK‐Blue hTLR2 cells. Now we identified, synthesized and developed a novel small molecule, SMU‐Z1, which can specifically activate TLR2. Our studies demonstrate that SMU‐Z1 specifically simulates TLR1 and TLR2 through the NF‐κB pathway to trigger the synthesis of proinflammatory cytokines, such as TNF‐α, IL‐1β, IL‐6 and nitric oxide. In addition, target‐protein based biophysical assays demonstrate that SMU‐Z1 possesses highly potent pharmacological characteristics. Furthermore, SMU‐Z1 promotes splenocyte proliferation, up‐regulates the expression of CD8^+^ T, NK and DC cells, which in turn resulted in antitumor immunity against leukemia in vivo. During our ongoing research period, the Nobel Prize winner Bruce Beutler et al. developed an interesting specific TLR1/2 small molecule agonist Diprovocim, which has synergy with PD‐L1 antibody to eliminate melanoma in a mouse model.^22^

By screening a synthetic library of 14 000 compounds (Maybridge) and a series of undisclosed compounds, we identified a class of compounds with a capacity for activating NF‐κB activation using HEK‐Blue hTLR2 cells (**Figure** **1**A,B). SMU‐Z1 was developed from this class of NF‐κB activations after extensive structure–activity relationship (SAR) studies. SMU‐Z1 induces NF‐κB activation in HEK‐Blue hTLR2 cells in a dose‐dependent manner with a EC_50_ of 4.88 ± 0.79 × 10^−9^ m, which is comparable to Pam_3_CSK_4_ (EC_50_ of 2.22 ± 0.23 × 10^−9^ m) positive control (Figure 1C; for the representative SAR results and discussion, see Tables S1 and S2, Supporting Information; for design, syntheses and compound characterizations, see Notes S1 and S2, Supporting Information).

**Figure 1:**
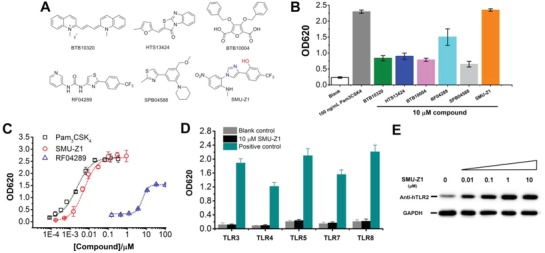
Structures of the hit compounds obtained from HTS, and bioactivity validation of the optimized compound SMU‐Z1. A) Chemical structures of the hits from the HTS. B) The initial hit compounds obtained for TLR2 activation. HEK‐Blue hTLR2 cells were incubated with indicated compounds for 24 h, and the cell culture supernatants were detected by QUANTI‐Blue (Invivogen) to evaluate SEAP signaling at 620 nm (OD620). Pam_3_CSK_4_ was used as a positive control in the experiment. C) Comparison of SMU‐Z1 with RF04289 and Pam_3_CSK_4_ for the SEAP activation in HEK‐Blue hTLR2 cells. D) HEK‐Blue human TLR3, TLR4, TLR5, TLR7, and TLR8 cells were incubated with SMU‐Z1 (10 × 10^−6^ m) with the control of TLR‐specific agonists separately for 24 h, and the activation was evaluated by QUANTI‐Blue at OD620. As positive control, agonists that selectively activate a specific TLR were used: TLR3, Poly I:C (10 µg mL^−1^); TLR4, LPS (10 ng mL^−1^); TLR5, FLA‐BS (1 µg mL^−1^); TLR7 and TLR8, R848 (5 µg mL^−1^). E) SMU‐Z1 stabilized more TLR2 protein in HEK‐Blue hTLR2 cells as the concentrations increase in the cultures. Cells were treated with SMU‐Z1 (0–10 × 10^−6^ m) for 24 h, and the cell lysates were detected using Western blot and rabbit anti‐TLR2 antibody. GAPDH served as the cell lysates input control. Data presented are mean ± SD and the figures shown are representative of three independent experiments.

To test whether the agonist SMU‐Z1 can specifically activate the TLR2 signaling pathway, HEK‐Blue hTLRs cells transfected with SEAP reporter genes were used. As shown in Figure 1D, SMU‐Z1 can specifically activate TLR2 but no other TLRs, including TLR3, TLR4, TLR5, TLR7, and TLR 8. Next, we tested whether SMU‐Z1 can bind to TLR2 and induce its expression directly. We observed that the synthesis of stabilized TLR2 protein was gradually increasing as we increased the dose of SMU‐Z1, which demonstrate SMU‐Z1 acts on TLR2 signaling pathway in a dose‐dependent manner within whole‐cell environment. However, HEK‐Blue hTLR2 cells can also endogenously express both TLR1 and TLR6, therefore whether SMU‐Z1 is TLR1/2 or TLR2/6 activator still remains unclear.

To determine whether SMU‐Z1 is a TLR1/2 or TLR2/6 agonist, we performed an antibody‐based selectivity assay. SMU‐Z1 efficiently activates about 95% SEAP signaling at 30 × 10^−9^ m. Addition of anti‐hTLR1 and anti‐hTLR2 but not anti‐hTLR6 antibodies changed such activation in a dose‐dependent way (**Figure** **2**A). To confirm whether the anti‐hTLR6 antibodies are functional, we used a TLR2/6 agonist Pam_2_CSK_4_, and tested the secreted embryonic alkaline phosphatase (SEAP) activation in the same cell line. We found that both anti‐hTLR2 and anti‐hTLR6 antibodies can restrain Pam_2_CSK_4_‐induced SEAP signaling, whereas anti‐hTLR1 antibodies did not block this activity (Figure 2B). In summary, we conclude that SMU‐Z1 primarily activates TLR1/2 but not TLR2/6 signaling.

**Figure 2:**
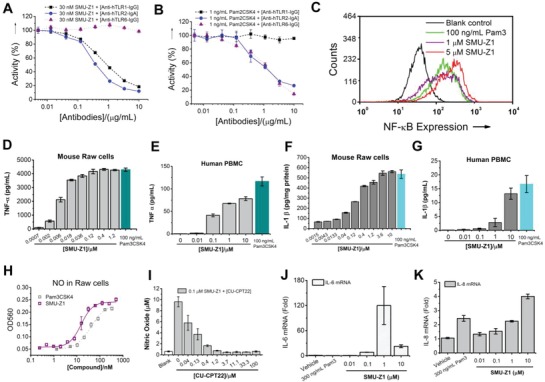
SMU‐Z1 is a TLR1/2, but not TLR2/6 agonist. A) HEK‐Blue hTLR2 cells were treated with SMU‐Z1 and anti‐hTLR1, anti‐hTLR2, or anti‐hTLR6 antibodies for 24 h. SMU‐Z1 strongly activates QUANTI‐Blue SEAP signaling at 30 × 10^−9^ m. Anti‐hTLR1‐IgG and anti‐hTLR2‐IgA antibodies inhibit SMU‐Z1‐triggered SEAP signaling in a dose‐dependent manner, whereas anti‐hTLR6‐IgG has no such influence. B) The positive control Pam_2_CSK_4_, a TLR2/6 agonist, had different responses to TLR1, 2 and 6 specific antibodies compared to SMU‐Z1. C) Flow cytometric analysis for 0 × 10^−6^, 1 × 10^−6^, and 5 × 10^−6^ m of SMU‐Z1 and Pam_3_CSK_4_. Results demonstrate excellent NF‐κB activation capacity of SMU‐Z1 compared to the positive control Pam_3_CSK_4_. D–G) SMU‐Z1 has stimulated D,E) TNF‐α and F,G) IL‐1β production in Raw 264.7 macrophage cells and human PBMC, respectively. H) SMU‐Z1 and Pam_3_CSK_4_ activate nitric oxide (NO) production in Raw cells in a dose‐dependent manner. I) NO activation of SMU‐Z1 can be inhibited by the TLR1/2 inhibitor, CU‐CPT22, in Raw 264.7 cells. SMU‐Z1 stimulates J) IL‐6 and K) IL‐8 mRNA production at different concentrations in human PBMC. Data are present as mean ± SD of triplicates and the results are representative of three independent experiments.

In order to investigate the cellular and molecular mechanisms of SMU‐Z1 actions, we developed a TLR2‐sensitive U937 human macrophage cell line with a GFP‐labeled NF‐κB reporter. Flow cytometry experiments demonstrated that SMU‐Z1 has activated NF‐κB signaling in a dose‐dependent manner. At the dose of 1 × 10^−6^ m, SMU‐Z1 showed comparable activation to 100 ng mL^−1^ of Pam_3_CSK_4_. At a higher dose (5 × 10^−6^ m), SMU‐Z1 had an even better effect (Figure 2C). Triptolide, a known NF‐κB inhibitor, was able to efficiently inhibit the SMU‐Z1 induced SEAP signaling (Figure S1, Supporting Information), which further implies that SMU‐Z1 works through the NF‐κB signaling pathway. To characterize the immunostimulatory activities of SMU‐Z1, Raw 264.7 macrophage cells and healthy human peripheral blood mononuclear cell (PBMC) were used. SMU‐Z1 stimulates a strong TNF‐α and IL‐1β production in a dose‐response manner (1 × 10^−9^ to 1 × 10^−6^ m) in both mouse cells (Figure 2D,F) and human PBMC cultures (Figure 2E,G). SMU‐Z1 stimulates NO synthesis^23^ similar to the TLR1/2‐specific agonist, Pam_3_CSK_4_ (Figure 2H). Furthermore, this NO production was suppressed by the known TLR1/2 inhibitor CU‐CPT22^20^ (Figure 2I). We have also confirmed the effects of SMU‐Z1 on mRNA expression of various proinflammatory cytokines by qRT‐PCR. The results revealed that the IL‐6 mRNA (Figure 2J) has significantly increased after treatment with 0.01 × 10^−6^ to 10 × 10^−6^ m of SMU‐Z1 in human PBMC, for example 1 × 10^−6^ m of SMU‐Z1 upregulated IL‐6 synthesis about 100 fold compared to the vehicle control. Similarly, IL‐8 mRNA (Figure 2K) has also increased about fourfold after treating PBMC with 10 × 10^−6^ m of SMU‐Z1. Collectively, our data suggest SMU‐Z1 as an efficient TLR1/2 agonist that can induce strong proinflammatory signals at both transcriptional and translational levels in mice and human cells by activating the NF‐κB pathway.

Above experiments suggest that SMU‐Z1 functions as an agonist of TLR1/2 heterodimer that can activate and promote the production of various downstream proinflammatory cytokines. To investigate whether SMU‐Z1 can directly bind to the TLR1 and TLR2 proteins, we biotin‐labeled the SMU‐Z1, and synthesized the compounds **45**, **46**, **47**, and **48**. Among them the compound **45** showed the best TLR1/2 activation effects (Table S2, Supporting Information, **Figure** **3**A), and was thus selected for subsequent biophysical studies. TLR1 and TLR2 was expressed and purified as previously reported.^20^ First, we immobilized human TLR1 proteins on the surface of streptavidin coated plates. Then, various concentrations of biotinylated‐SMU‐Z1 (compound **45**) were added, and the bound compound **45** was detected by streptavidin‐horseradish peroxidase (HRP) and TMB substrate solution. As shown in Figure 3B, biotinylated SMU‐Z1 bound to TLR1 in a concentration‐dependent manner with little binding to the control, BSA, indicating the specific binding of SMU‐Z1 to TLR1 protein. Interestingly, Pam_3_CSK_4_ was found to compete with immobilized SMU‐Z1 for binding to TLR1 (Figure 3C). Based on this result, we did the binding assay of SMU‐Z1 to TLR2 and the results showed that it binds to human TLR2 in a concentration‐dependent manner similar to TLR1 (Figure 3D); Pam_3_CSK_4_ was also found to compete with immobilized SMU‐Z1 for binding to TLR2 as well (Figure 3E). These results demonstrate that SMU‐Z1 functions similar to Pam_3_CSK_4_ by binding to TLR1 and TLR2 to trigger immune responses, which is consistence with the observation in the whole cell assay.

**Figure 3:**
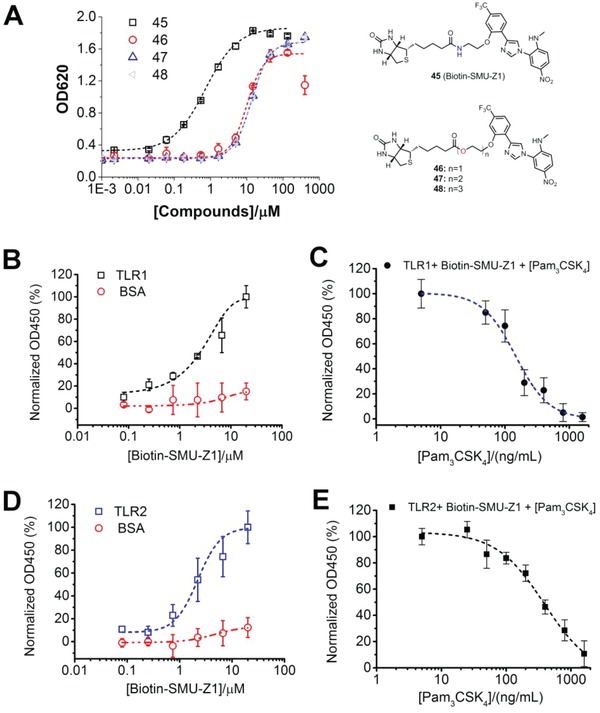
Biophysical characteristics of SMU‐Z1 binding to TLR1 and TLR2. A) Comparison of biotin‐labeled compounds 45 to 48 to induce SEAP gene expression. HEK‐Blue hTLR2 cells were incubated with the indicated concentrations of compounds 45 to 48 for 24 h, and the activation was measured by Quati‐Blue reagent to detect the SEAP signaling in the culture supernatants at OD620. B) Human TLR1 (5 µg mL^−1^) or BSA (5 µg mL^−1^) was added to the streptavidin precoated plate. Then different concentrations of biotin‐labeled SMU‐Z1 were added, and the bound biotin‐SMU‐Z1 was determined by streptavidin‐horseradish peroxidase (HRP). Absorbance with TLR1 in the presence of 20 × 10^−6^ m Biotin‐SMU‐Z1 was set as 100%. C) Human TLR1 (5 µg mL^−1^) was added to the streptavidin pre‐coated plate. Biotin‐SMU‐Z1 (10 × 10^−6^ m) and different concentrations of Pam_3_CSK_4_ were added, and the bound SMU‐Z1 was detected by streptavidin‐HRP. Absorbance with 0 ng mL^−1^ of Pam_3_CSK_4_ was set as 100%. D) Human TLR2 (5 µg mL^−1^) or BSA was added to the streptavidin precoated plate. Then different concentrations of biotin‐labeled SMU‐Z1 were added, and the bound biotin‐SMU‐Z1 was determined by streptavidin‐HRP. Absorbance with TLR2 in the presence of 20 × 10^−6^ m of Biotin‐SMU‐Z1 was set as 100%. E) Human TLR2 (5 µg mL^−1^) were added to the streptavidin precoated plate and the other experimental methods are the same as that in Figure 3C. Data are present as mean ± SD of triplicates and the results are representative of three independent experiments.

With the excellent simulation of immune responses in cells and having target specificity in biophysical experiments, we explored the anti‐tumor immunity of SMU‐Z1 both in vitro and in vivo. Mouse primary splenocytes are mainly composed of B and T cells, and may contain about 10% antigen‐presenting cells (APCs) as well as other cells.^24^ In this study, the effects of SMU‐Z1 on splenocyte proliferation and their viability were measured by CCK8 after treatment with SMU‐Z1 for 48 h (**Figure** **4**A) in male C57Bl/6 (C57) mice. The results demonstrate that splenocyte proliferation has significantly and optimally enhanced three to fivefold by 5 × 10^−6^ m of SMU‐Z1 after 2 d (Figure 4B). These proliferation results motivated our interest to investigate the potential antitumor immunity of SMU‐Z1 in vivo. The proliferation of spleen cells potentially reflected an increase in B or T cells. In order to specifically clarify the proliferation of B cells and effector T cells in vivo, we used flow cytometry for analysis on two different days (days 5^21^ and 7). CD19^+^, CD3^+^, CD4^+^, CD8^+^, and TLR2^+^ markers were used. C57 mice were immunized by intraperitoneal injection of SMU‐Z1 (0.1 mg per mice), Pam_3_CSK_4_ (5 µg per mice^21^) or PBS for 5 to 7 d. After sacrifice, the percentage of positive cells in spleen, i.e., CD19^+^ B cells, CD4^+^ and CD8^+^ T cells, were determined by flow cytometry. No significant difference between the control and treated mice was observed in B cell numbers (Figure 4C). However, CD8^+^ T cells showed a significant increase, from 17.1 ± 0.4% to 27.4 ± 0.3% in the control and SMU‐Z1 treated mice after 7 d (Figure 4D). The increase in CD8^+^ cytotoxic T lymphocytes (CTLs) was comparable between Pam_3_CSK_4_ (20.8 ± 0.6%) and SMU‐Z1 (21.3 ± 0.2%) treated groups after 5 d. However, CD4^+^ T cells decreased after SMU‐Z1 (55.6 ± 0.7%) or Pam_3_CSK_4_ (65.0 ± 0.5%) treatment, when compared to PBS control (69.5 ± 1.3%) (Figure 4D). An increase in the CD8/CD4 ratio was observed (Figure 4D, right panel) after SMU‐Z1 treatment for 7 d. Further, we confirmed that the proliferation of CD8^+^ T cells is associated with TLR2 stimulation. PBS‐treated mice had 2.6 ± 0.1% of CD3^+^CD8^+^TLR2^+^ T cells, and the levels of this T‐cell population were dramatically increased after treatment with SMU‐Z1 on both day 5 (6.3 ± 0.7%) and day 7 (6.7 ± 0.2%) (Figure 4E). We observed that SMU‐Z1 has better TLR2 stimulation than Pam_3_CSK_4_ in these experiments. This might probably because of the ester bond of Pam_3_CSK_4_, which could be rapidly degraded in vivo. Breakdown of ester bonds connecting glycerol backbone and palmitic acid chains could be a plausible explanation for the low level of TLR2 activation by this agonist, Pam_3_CSK_4_. SMU‐Z1 also dose‐dependently activates CD4^+^ and CD8^+^ T cells in the isolate T cells. The proliferation percentage of CD8^+^ T cells (≈60%) is much higher than CD4^+^ T cells (≈35%), which indicate that SMU‐Z1 has a greater impact on CD8^+^ T cells (Figure S2, Supporting Information). Our result is consistent with an earlier report describing TLR2 involvement in the proliferation and survival of CD8^+^ T cells than CD4^+^ T cells.^25^ Since CD8^+^ CTLs are very important in tumor killing,^26^ we next evaluated the anti‐tumor immunity of SMU‐Z1.

**Figure 4:**
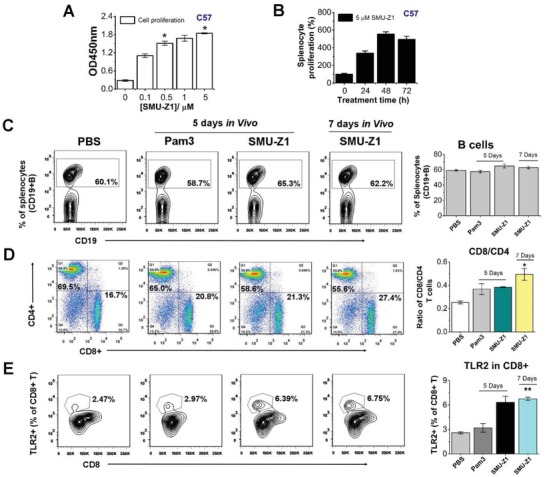
SMU‐Z1 increases splenocyte proliferation, CD8^+^/CD4^+^ ratio, and TLR2 expression. A) SMU‐Z1 treatment accelerated splenocyte proliferation at different concentrations. B) Time kinetics of SMU‐Z1 stimulated primary splenocyte proliferation at 5 × 10^−6^ m. Cell proliferation was tested by measuring CCK8 absorbance at 450 nm at different time intervals. C) Percentages of CD19^+^ B cells present within splenocytes were determined by flow cytometry at various time points after treatment with PBS, 5 µg Pam_3_CSK_4_ (Pam3), or 0.1 mg of SMU‐Z1 in C57BL/6 mice. D) Representative graphs of CD4^+^ and CD8^+^ T cells from PBS, Pam_3_CSK_4_ (Pam3), or SMU‐Z1‐treated mice. The percentage of CD8^+^ and ratio of CD8^+^/CD4^+^ T cells increased after treatment with SMU‐Z1. E) Percentage of TLR2 positive cells (CD3^+^CD8^+^TLR2^+^) in the spleen. Results shown are average values from three independent experiments, and the data are presented as mean ± SD. *, *p* < 0.01; **, *p* < 0.001 compared to the control group.

Previous reports suggest that TLR1/2 agonist BLP (bacterial lipoprotein) can effectively inhibit lung cancer, leukemia and melanoma development, leading to a long‐lasting protective response against tumor rechallenge.^11^ Moreover, TLR1/2's agonist PSK has shown significant effects in the inhibition of both implanted and spontaneous breast tumors and is already in phase II clinical trial.^2^ However, in vivo antitumor activity of TLR1/2 small molecule agonists has rarely been reported. We hypothesized that because of SMU‐Z1 promotion of the proliferation of CTLs, it can function as an antitumor agent. We tested this hypothesis and found that SMU‐Z1 increases the proliferation of CD8^+^ T cells, NK cells, DCs, and decreases the progression of leukemia tumor volume significantly compared to control mice. Groups of C57 mice were implanted with syngeneic murine FBL3 leukemia cancer cells (5 × 10^5^ cells per mice, 100 µL in PBS), on day 7 when tumors were palpable, mice were treated with SMU‐Z1 or PBS intraperitoneally for every 5 d. Tumor size and survival of the mice were monitored regularly. It was worth noting that after three administrations of 0.3 mg of SMU‐Z1, on day 23 (**Figure** **5**A), the tumors in three out of seven mice have completely disappeared. Splenocytes were collected 35 d after inoculation, and single‐cell suspensions were antibody stained and analyzed by flow cytometry to detect the total number of B cells, CD3^+^, CD4^+^ and CD8^+^ T cells, NK cells, and DCs. In the drug treatment group, representative results for tumor retraction and shrinkage are listed separately (Figure 5B–E). SMU‐Z1 has significantly increased the frequency of CD3^+^ T cells (25.7 ± 1.4%) in the spleen compared to the vehicle control (11.6 ± 0.5%) (Figure 5B). However, no significant difference in B cell numbers was observed (data not shown). Further analysis of these CD3^+^ T cells revealed that SMU‐Z1 when compared to controls, increased the frequencies of CD8^+^ T cells (19.4% vs 10.6%), as well as upregulated the CD8/CD4 ratio (0.28% vs 0.13%) (Figure 5C). SMU‐Z1 immunization has increased NK cells (Figure 5D) and DC cells (Figure 5E) as well. These observations illustrate that SMU‐Z1 has a strong antitumor potential. As shown in Figure 5F, SMU‐Z1 treatment has significantly inhibited the growth of leukemia tumor in C57 mice. The tumor size after 35 d of treatment was 2370 ± 372 mm^3^ in the control group and 386 ± 280 mm^3^ in the SMU‐Z1 group (Figure 5F). Thus, treatment with SMU‐Z1 has significantly reduced the tumor volume, but not the body weight of the mice (Figure 5G). These results demonstrate the significant anti‐tumor immunity of SMU‐Z1 against leukemia in vivo.

**Figure 5:**
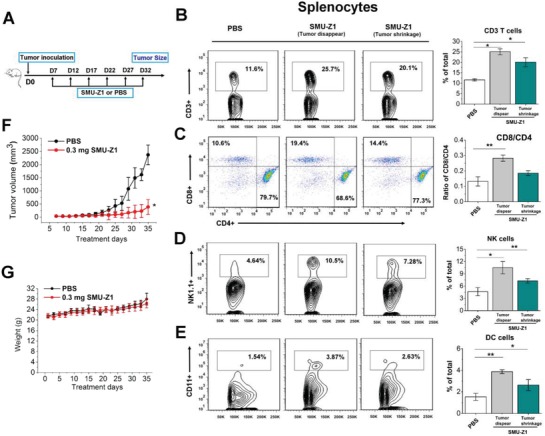
SMU‐Z1 enhances anti‐tumor CTL responses and inhibits the growth of implanted FBL3 leukemia cancer cells in vivo. A) Schematic diagram of treatment protocol. Eight weeks old male C57Bl/6 mice were inoculated with FBL3 cells (5 × 10^5^ cells per mice) for 7 d to establish the solid tumors. Mice were treated intraperitoneally with 100 µL of PBS, or SMU‐Z1 (0.3 mg in 100 µL of PBS) once in every 5 d. Splenocytes were harvested on day 35 to isolate and analyze the CTLs. Tumor volumes and body weight of individual groups of mice were monitored (*n* = 7 per group). B–E) Frequency of each cell type of splenocytes in PBS or SMU‐Z1 treated groups. In the SMU‐Z1 treatment group, data on tumor disappearance (three mice) and tumor shrinkage (four mice) are listed separately. B) Percentage of CD3^+^ T cells, C) CD4^+^ (CD4^+^CD3^+^) and CD8^+^ T cells (CD8^+^CD3^+^), D) NK cells (NK1.1^+^), E) DC cells (CD11c^+^) within splenocyte population. F) The volume of tumors on different treatment days is given. *p < 0.01 versus control at the last measurement day. G) The body weight of PBS or SMU‐Z1 treated mice. *p < 0.01, **p < 0.001. All the results are representative examples of three independent experiments, and the data are presented as mean ± SD.

We have also analyzed tumor infiltrating lymphocytes (TIL) and found significant differences between control and SMU‐Z1 treated mice. In the control group, lymphocytes in the tumor tissue were negligible. However, high percentage of lymphocytes were present in the SMU‐Z1 treated group (**Figure** **6**A). We have further analyzed the nature of TILs in the SMU‐Z1 treated group. The frequency of CD3^+^ T cells in the tumor tissue (30.0%) was higher than the frequency of these cells in the splenocytes (21.1%), but the CD8/CD4 ratio was comparable in both tissues. NK and DC cells were also present in the tumor tissue site (Figure 6A). TLR2 was found to be expressed on CD8^+^ T, NK1.1^+^ NK and CD11c^+^ DC cells in the SMU‐Z1 treated group. The frequency of TLR2 expression was relatively higher in CD8^+^CD3^+^ T cells (Figure 6A), which further indicate the possibility of TLR2 association with antitumor effect. Presented data support our hypothesized mechanism that SMU‐Z1 binds to TLR1/TLR2 present on APC, triggers the activation costimulatory molecules, leading to promotion of cytokines secretion, which in turn leads to activation and proliferation of CD4^+^ and CD8^+^ T cells. CTLs thus generated induce anti‐tumor immunity against leukemia cells, including induction of NK‐mediated tumor cell lysis (Figure 6B).

**Figure 6:**
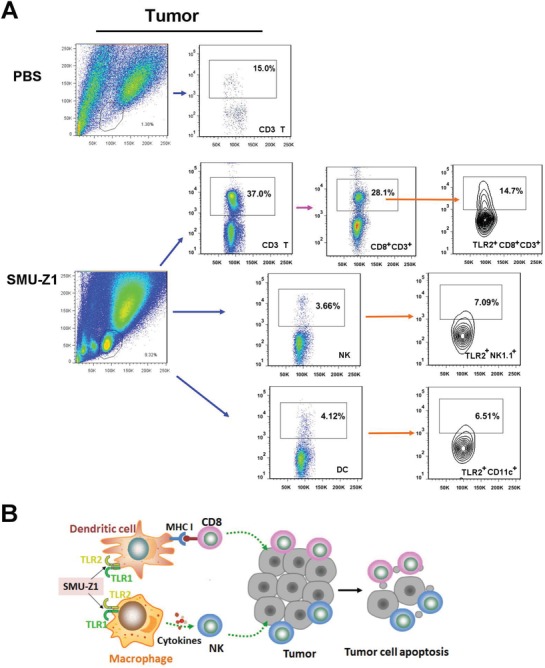
SMU‐Z1 increases tumor infiltrating lymphocytes. A) Similar to experiment described in Figure 5, tumors were harvested on day 35 to isolate and analyze TILs. Representative flow graph shows frequency of each types of TILs in PBS or SMU‐Z1 treated groups, including CD3^+^ T, CD8^+^CD3^+^ T, CD4^+^CD3^+^ T, TLR2^+^CD8^+^CD3^+^, TLR2^+^CD4^+^CD3^+^, NK, TLR2^+^NK1.1^+^, DC, TLR2^+^CD11c^+^ cells. B) Model of key cellular events mediating the anti‐tumor effect of SMU‐Z1. All the results are representative of two independent experiments.

Selective targeting of the TLR family proteins has generated great interest in wide variety of research fields, but developing drug‐like, small molecule compounds with high affinity and selectivity to TLRs is a difficult task. Targeting the TLR1/2 complex is particularly challenging because of the high dynamic nature of ligand‐binding pockets present at protein–protein interfaces.^27^ Since the protein–protein interactions (PPI) are usually extensive, there is a requirement for large ligands to interfere with their functions more effectively. All the current TLR2 agonists, such as Pam_3_CSK_4_ for TLR1/2 and Pam_2_CSK_4_ or FSL‐1 for TLR2/6, are primarily synthetic lipoproteins containing two or three 15‐carbon acyl chains with molecular weights ≈1500 Da, which hinder their further development into optimal therapeutics. Secondly, TLR selectivity poses a great challenge for small molecule agonists and antagonists as there are at least 13 homologous TLRs in rodents and 10 in humans, having similar structures. This is especially the case for TLR1/2 and TLR2/6, because of the heterodimeric protein complexes that share one common TLR2 protein. A third challenge in developing TLR agonists is that native TLR ligands are often highly toxic, which significantly limit their applications. The currently available TLR2 agonists have limited applications due to the ester linkages, which easily can undergo hydrolysis by esterases present in the plasma or be rapidly degraded by gastric acids present in the stomach, resulting in diminished stimulation of TLR2. Compared to the existing TLR2 lipopeptide activators or polysaccharide, small molecule agents have the merits of not easily prone to hydrolysis, low cost of production, high productivity yields, increased tissue permeability characteristics but without compromising immunogenic properties.

In this report, we have successfully developed a highly potent, low toxicity (Figure S3, Supporting Information) and selective small molecule agonist of TLR1/2 with an EC_50_ value of 4.88 ± 0.79 × 10^−9^ m. Cellular studies demonstrate that SMU‐Z1 activates HEK‐Blue cells that are overexpressing hTLR2, but not other TLRs. This activation can be blocked either by anti‐hTLR1 or anti‐hTLR2 antibodies, but not with anti‐hTLR6 antibodies, indicating that SMU‐Z1 has high selectivity for the TLR1/2 heterodimer. Furthermore, SMU‐Z1 can bind to TLR1 and TLR2 proteins and function similar to Pam_3_CSK_4_ in the biophysical assays. In addition, we show that SMU‐Z1 signaled through NF‐κB and increased the production of the downstream effector molecules, such as TNF‐α, IL‐1β, IL6, IL‐8 and NO. Importantly, SMU‐Z1 functions by promoting splenic proliferation, especially by up‐regulating the CD8^+^ cytotoxic T lymphocytes, NK cells and DC cells, which in turn contribute to significant anti‐tumor response against FBL3 leukemia in vivo. Our work, therefore, describes the development of a first well characterized, specific small molecule agonist for TLR1/2, and characterizes the possible mechanisms of SMU‐Z1 functions. This study could lead to potential therapeutic applications in developing new vaccine adjuvants and in developing new anti‐tumor immune therapies in the near future.

## Experimental Section

*Experimental Design*: Limitations of currently available TLR agonists promoted us to screening a library of compounds using HTS strategy, identify, design and synthesize 48 new small molecules specific to TLR2. Base on this intensive work, a best‐in‐class compound SMU‐Z1 with a EC_50_ of 5 × 10^−9^ m was developed. The specificity was verified by various TLR transporter cells. The signaling pathway was confirmed in the GFP‐labeled NF‐κB reporter U937 cell line. Human PBMC and Raw 264.7 cells were employed for the downstream cytokines evaluation. Human TLR1 and TLR2 protein were expressed in insect H5 cells for the biophysical target verification. The animal experiments conformed to the Southern Medical University's Committee on the Care and Use of Laboratory Animals Guidelines.

*QUANTI‐Blue SEAP Assay*: Cells were cultured in 96‐well plates (4 × 10^4^ cells per well) of 200 µL Dulbecco's modified eagle medium (DMEM) (supplemented with 10% fetal bovine serum (FBS), 10 × penicillin/streptomycin, and 10 ×l‐glutamine) at 37 °C for 24 h before drug treatment on the first day. In the next 24 h of treatment, medium was removed from the 96‐well plate and substituted with 200 µL of DMEM containing indicated concentrations of compounds, antibody, or ligands in the half maximal effective concentration (EC_50_) determinations. Different antibodies (0–10 µg mL^−1^), including anti‐hTLR1‐IgG, anti‐hTLR2‐IgA, or anti‐hTLR6‐IgA (InvivoGen) were used in the antibody experiments. Different TLRs ligands was used as positive control in the specificity assays, such as Pam_3_CSK_4_ for TLR1/2, poly(I:C) (10 µg mL^−1^) for TLR3, LPS (10 ng mL^−1^) for TLR4, FLA‐BS (1 µg mL^−1^) for TLR5, and R848 (5 µg mL^−1^) for TLR7 and TLR8. A sample buffer (50 µL) was collected and transferred from each well of the cell culture supernatants to a transparent 96‐well plate (Thermo Scientific). Each well was treated with 50 µL of QUANTI‐Blue (InvivoGen) buffer and incubated at 37 °C for 0.5‐1 h. Then measure the purple color by using a plate reader at an absorbance of 620 nm (A620).

*U937 Cell Transfection and NF‐kB‐GFP Reporter Assay*: Human macrophage U937 cells [American Type Culture Collection (ATCC) CRL‐1593.2] were cultured in RPMI 1640 medium (supplemented with 10% FBS, 1% pen/strep). By using the commercially available pGreenFire plasmid (System Biosciences) was stably inserted an NF‐κB‐GFP reporter. Briefly, HEK 293T cells (ATCC CRL‐3216) were transfected by using a polyethylenimine/DNA ratio of 6:1 accompanied with the pGreenFire vector (4.33 µg) and the pREV (4.33 µg), pMDL (4.33 µg) and pVSVg (2 µg) viral packaging plasmids. Then harvest viral particles from the medium after 48 to 72 h transfection and concentrated by using an 8.5% PEG‐8000 (polyethylene glycol, molecular weight 8000) and 10 × 10^−3^ m NaCl solution. The concentrated virus and polybrene (8 µg mL^−1^) were added to U937 cells for 48 h. Then, select the stably transfected cells by using U937 growth medium supplemented with puromycin (1 mg mL^−1^). After selection process finished, using a MoFlo Cytomation (Beckman Coulter) fluorescence‐activated cell sorter to sort the cells for GFP expression. After sorting for insertion, cells were treated with a TLR1/2 agonist Pam_3_CSK_4_ [66 × 10^−9^ m (100 ng mL^−1^), InvivoGen] and sorted for activation. Then between the untreated and the treated cells the top 10% of activated cells were collected for each sort until no further peak separation was achieved. The sorted cells were seeded in six‐well plates at density of 1 × 10^6^ cells per well with 3 mL growth medium of RPMI 1640 medium [supplemented with 10% FBS, penicillin (100 U mL^−1^), and streptomycin (100 mg mL^−1^)] and the indicated concentrations of compound and Pam_3_CSK_4_ for 24 h at 37 °C in a 5% CO_2_ humidified incubator. 24 h later before the flow cytometry analysis, the cells of each well were mixed and then use propidium iodide to stain 200 µL of cells containing medium for 10 min.

*Griess for Detecting Nitric Oxide in Raw 264.7 Cells*: The standard nitrite of NO_2_^−^ solution was prepared base on the concentration range of standard curve. Dissolving 85% strong phosphoric acid (6 mL) and sulfanilic acid (1.0 g) in water to make 100 mL obtained substrate solution A, while dissolving naphthyl ethylenediamine dihydrochloride (0.1 g) in water to make 100 mL obtained substrate solution B. On the first day, Raw 264.7 cells (mouse leukemic monocyte macrophage cell line) were cultured in medium of RPMI 1640 supplemented with 10% FBS, and 1% pen/strep, seeded in 96‐well plates at 80 000 cells of density per well, and preincubated for 24 h at 37 °C in 5% CO_2_ humidified incubator. Then replaced the media with DMEM only and added different concentration of compounds or Pam_3_CSK_4_ with 200 µL volume totally, and it was incubated for another 24 h. After incubation, 50 µL of standard nitrite solution and cell supernatants were added to 96‐well‐flat microplate (Thermo Scientific), then added 50 µL substrate solutions A and incubated for 10 min. After that, another 50 µL substrate solution B was added and incubated in 37 °C incubator for 10 min.^28^ Ultimately, absorbance was detected at 560 nm by a microplate reader (Thermo Scientific) and afforded the content of nitric oxide by Origin 9.0.

*ELISA Assay*: *TNF‐α and IL‐1β ELISA*: TNF‐α and IL‐1β assay kits (Mouse, BD Biosciences) were used according to the manufacturer's instructions. Raw 264.7 cells were cultivated in six‐well plates (Thermo Scientific) at density of 1 × 10^6^ cells per well with 3 mL medium of RPMI 1640 [supplemented with 10% FBS, 1% pen/strep] and incubate for 24 h at 37 °C in a 5% CO_2_ humidified incubator. After 24 h, nonadherent cells and medium were removed and replaced with unsupplemented fresh RPMI 1640 medium (3 mL per well). The cells were treated with indicated concentrations of SMU‐Z1 and the positive control was 100 ng mL^−1^ of Pam_3_CSK_4_ (InvivoGen). Plates were then incubated for another 24 h, and then collected and froze cell culture supernatants at −80 °C until ready for measuring the cytokine production. Using cytokine specific capture antibodies, detection antibodies, and recombinant human cytokine to quantify TNF‐α or IL‐1β production. The cytokine level in each sample was conducted in triplicate assays.

*Cytokines in PBMC*: PBMCs were seeded in 12‐well plates at a density of 2.5 × 10^5^ cells per well with 0.5 mL of medium [supplemented with 10% FBS, 1% pen/strep]. The cells were treated with indicated concentration of SMU‐Z1 and incubated for 24 h at 37 °C in a 5% CO_2_ humidified incubator. The cell culture supernatants were collected and froze at −80 °C until measurement. The level of cytokine TNF‐α or IL‐1β was determined using recombinant cytokine standards, cytokine‐specific capture antibodies, and detection antibodies according to the commercially available human ELISA kit (BD Biosciences) with each sample for duplicate.

*Streptavidin Precoated Plate*: pH 5.0, 0.15 m of citric acid‐phosphate buffer solution were prepared from the mixture of citric acid (1030 mL, 0.1 m) and Na_2_HPO_4_ (970 mL 0.2 m) (Sigma‐Aldrich). Streptavidin (Sigma‐Aldrich) was added to the buffer solution to the concentration of 2 µg mL^−1^ and afforded coating buffer. Each well of Nunc 96 well (Thermo Scientific) was added 100 µL of citric acid–phosphate buffer solution and incubated in temperature of 37 °C of air dry oven over night until dry and then afforded streptavidin pre‐coated plate. Finally, sealing the Nunc 96‐well plate and placed under 4 °C before use.

*Competition Binding ELISA Assay*: A mixture of human TLR1 (5 µg mL^−1^) or TLR2 (5 µg mL^−1^) or BSA (5 µg mL^−1^) was coated on the streptavidin pre‐coated Nunc 96‐well ELISA plates in acetate buffer solution (pH 5.0) at room temperature for 2 h as the capturing probe. The wells were washed three times with PBST buffer (PBS supplemented with 0.05% Tween‐20) and then blocked with a 5% BSA solution at room temperature for 1 h. After washing with PBST three times, the indicated concentration of biotin‐labeled SMU‐Z1 was added and incubated for 1 h at room temperature. After five washings with PBST, streptavidin‐coupled HRP conjugate was diluted at a ratio of 1:2000 and added into the wells, and then incubated at room temperature for 1 h. After washing with PBST seven times, each well was added 100 µL of TMB reagents (BD OptEIA) and incubated at room temperature for 10–30 min. Fifty microliters of 50 µL 1 m H_3_PO_4_ was subsequently added into each well to stop the reaction, measured by multiscan FC microplate reader (Thermo Scientific) for 450 nm absorbance.

*Cell Viability Assay*: HEK‐hTLR2 cells (3 × 10^4^ cells per well) or Raw 264.7 cells (2 × 10^4^ cells per well) were seeded in 96‐flash plate with 100 µL DMEM medium (with 10% FBS and 1% pen/strep) and incubated at 37 °C overnight. After that, indicated concentration compounds of SMU‐Z1 were added to 200 µL totally and incubated at 37 °C for another 24 h. After 100 µL supernatant was removed, 10 µL Cell counting kit‐8 (CCK‐8) (Bimake, B34304, USA) was added into each well of above 96‐well plate and incubated at 37 °C for 1–4 h until it turned into orange. Then the plate was measured at an absorbance of 450 nm through a plate reader.

*Splenocytes Proliferation Assay*: Mouse spleens were isolated from C57Bl/6 mice aged from 8–10 weeks in each group under aseptic conditions. After treatment with lymphocyte lysis buffer, the splenocyte dispersion was homogenized and filtrated through a 300‐mesh sieve, then separated under differential centrifugation (Thermo Scientific). The cells were harvested and resuspended with RPMI 1640 medium twice to obtain single cell suspensions. 3 × 10^5^ cells were seeded each well of a 96‐well‐flat bottomed microplate and treated with indicated concentration of SMU‐Z1 to totally 200 µL, then routinely grown 24, 48, and 72 h at 37 °C in a humidified atmosphere of 5% CO_2_ incubator. Subsequently, cell counting kit‐8 (Dojindo; Japan) solution was added according to manufacturer instruction and detected the absorbance at 450 nm in a microplate reader (Thermo Scientific) according the manufacturer's instruction.

*Mice Peripheral Blood Leukocytes and Splenocytes Separation and Staining*: C57Bl/6 male mice were randomly divided into three groups (*n* = 3 each) and were administered with 0.1 mg of SMU‐Z1 or vehicle by intraperitoneal injection. For peripheral blood leukocyte, the blood from the orbital venous was harvested in heparinized tube at days 5 and 7 after dosing. Red blood cell lysis buffer was added in a proportion of 1:5 according to the manufacturer's instruction (BD, 555899). After gently vortex, the tube was incubated for 15 min at room temperature in dark and then carefully aspirated the supernatant after centrifugation at 200 g for 5 min. The cells were washed twice with PBS (with 0.5% BSA and 0.2% 1 × 10^−6^ m EDTA). For spleen leukocyte, the mouse spleen was harvested, minced, and filtered to prepare a single cell suspension. And 2 mL red blood cell lysis buffer was added and incubated for 5 min at room temperature in dark with occasional shaking. Then the supernatant was carefully aspirated after 300 g for 5 min and the cells were washed twice with PBS. After pelleting, mouse peripheral blood and spleen leukocyte were stained with a mixture of antibodies for 30 min, including anti‐mouse CD19‐APC (BD, 561738), anti‐mouse CD3e‐APC‐Cy7 (BD, 561 042), antimouse CD8a‐V500 (BD, 560 778), anti‐mouse CD4‐PE(BD, 553 048), antimouse NK‐1.1‐PerCP‐Cy5.5 PK136 (BD, 56 111), antimouse CD11c‐PE HL3 (BD, 561 044), anti‐mouse CD11b‐FITC (BD, 557396), antimouse F4/80‐PE (BD, 565410) and FITC CD282 (TLR2) monoclonal antibody (6C2) (eBioscience, 11‐9021‐80). After the incubation, the tube was centrifuged at 900 rmp for 5 min and washed twice with the PBS. Stained cells were analyzed with flow cytometer (FACScanto II, BD) and the flow cytometry data were analyzed using FlowJo software.

*In Vivo Antitumor Effects of SMU‐Z1*: Male C57BL/6 mice at 8 weeks of age (17–19 g) were obtained from Southern Medical University. Before the beginning of the study, the mice were allowed to adapt to the environment for 7 d. All of mice were raised under standard conditions with a 12 h light‐dark cycle at 22 ± 1 °C and 55 ± 5% humidity with food and water provided ad libitum. Male C57BL/6 mice were inoculated subcutaneously with 5 × 10^5^ FBL3 leukemia cancer cells. After the formation of a solid tumor with a volume of about 50 mm^3^, the tumor‐bearing mice were randomly divided into two groups with seven mice in each group. Then treatment with groups of mice by PBS or SMU‐Z1 (0.3 mg, i.p.) once every 5 d. At the end of the experiment, the mice were sacrificed. The tumor diameters were measured with calipers, and the tumor volume was calculated by the formula *V* (mm^3^) = 0.5326 × length × width × hight.^9^

*qRT‐PCR*: PBMC cells were seeded at a density of 1 × 10^6^ cells per well with 3 mL of medium [RPMI 1640 medium supplemented with 10% FBS and 1% pen/strep] in six‐well plates. The cells were treated with the indicated concentrations of SMU‐Z1 and positive control of 300 ng mL^−1^ Pam_3_CSK_4_ and grown for 24 h at humidified incubator of 37 °C and 5% CO_2_. Then, the medium was removed by centrifuge at 800 rpm and gently washed the cells with cold PBS (3 × 1 mL). Then, 1 mL of PBS was added to each sample. The mixture was transferred into corresponding 1.5 mL cryotubes and frozen at the range of −70 °C to −80 °C until ready for qRT‐PCR. RNeasy Mini Kit (SABiosciences) was used to extract the total RNA according to the manufacturer's instruction steps. Complementary DNA (cDNA) was synthesized by RT2 Easy First Strand cDNA Synthesis Kit (SABiosciences) according to the manufacturer's instruction. The primers for IL‐6, IL‐8, and GAPDH were purchased from SABiosciences. qPCR was performed on a CFX96 Real‐Time PCR detection system (Bio‐Rad) using the SYBR Green method. The data were analyzed by ΔΔ*Ct* method.

*Western Blotting Analysis*: In the first day, 3 mL of HEK‐Blue hTLR2 cells was seeded in six‐well plate (Thermo Scientific) at density of 1.5 × 10^6^ per well in 3 mL culture medium (DMEM supplemented with 10% FBS, 10 × penicillin/streptomycin, and 10 × L‐glutamine) and incubated for 24 h. Next, the medium was replaced with 3 mL culture medium, and cells were stimulated with indicated concentration of SMU‐Z1 or Pam3CSK4 for another 24 h. Cells were harvested and lysed with 150 µL cell lysis buffers (PIPA mixed with PMSF at ratio of 50:1, Boster) for 30 min on ice. After quantification and denaturation, cell lysates were separated by 10% sodium dodecyl sulfate polycrylamide gel electrophoresis (SDS/PAGE) and transferred to polyvinylidene fluoride (PVDF) membrane (Millipore, IPVH00010). Nonspecific reactivity was blocked in TBST with 5% skim milk (BD, 6342932) for 1 h at room temperature. Membranes were then probed with the primary antibodies overnight at 4 °C and secondary antibodies at room temperature for 1 h. Reactive protein was detected by ECL chemiluminescence system (ProteinSimple, FlourChem R). During the operation, the following primary and secondary antibodies were employed: mouse‐GAPDH (1:2000, Solarbio, M1000110), rabbit‐TLR1 (1:2000, Cell Signaling, 2209S), rabbit‐TLR2 (1:2000, Cell Signaling, 12276S), goat‐anti‐mouse‐HRP (1:2500, Boster, BA1050) and goat‐anti‐rabbit‐HRP (1:1000, Solarbio, SE134).

*Measurement of Mouse T Cells Cytokine Production and T‐Cell Proliferation*: T cells were isolated from mouse spleen by a mouse Pan naïve T cells negative selection kit (19848A, Stem cell). To detect the proliferation of CD4^+^ T cells and CD8^+^ T cells, the isolated cells were firstly stained with 0.4 µm CFSE for 10 min at room temperature. And then the cells stimulated with 5 µg mL^−1^ plate‐bound anti‐CD3 (BE0001‐1‐A005MG, BioXcell) and anti‐CD28 antibodies (BE0015‐1‐A005MG, BioXcell) for 48 or 72 h and incubated in 1 mL proliferation culture medium (1640 + 10% FBS+βME+10 ng mL^−1^ IL‐2) containing 0 × 10^−9^, 10 × 10^−9^, 100 × 10^−9^, 1000 × 10^−9^ m SMU‐Z1. After 48 or 72 h, cells were collected and stained with eFluor 660‐conjugated anti‐CD4 (50‐0041‐82, eBioscience) and PE‐Cy7‐conjugated anti‐CD8α (25‐0081‐82, eBioscience). Then flow cytometry was used to measure T‐cell proliferation by analyzed the percentage of CFSE^low^ T cells.

*Statistical Analysis*: All data from cell culture experiments were performed on the basis of three individual cell preparations unless otherwise noted. Data are expressed as mean ± standard deviation (SD). Statistical significances were determined with use of the unpaired Student's t test. Values of *p* < 0.05 were considered as statistically significant.

## Conflict of Interest

The authors declare no conflict of interest.

## Supporting information

Supplementary
