# Supplementary material for: TLR1/2 Specific Small‐Molecule Agonist Suppresses Leukemia Cancer Cell Growth by Stimulating Cytotoxic T Lymphocytes
Source: Adv Sci (Weinh). 2019 Mar 27;6(10):1802042. doi: 10.1002/advs.201802042 (PMC6523386; doi:10.1002/advs.201802042)
Supplement: Supplementary file 1 — Supplementary [file ADVS-6-1802042-s001.pdf]

Copyright WILEY-VCH Verlag GmbH & Co. KGaA, 69469 Weinheim, Germany, 2019.

ADVANCED SCIENCE
Open Access

Supporting Information

for *Adv. Sci.*, DOI: 10.1002/advs.201802042

TLR1/2 Specific Small-Molecule Agonist Suppresses Leukemia Cancer Cell Growth by Stimulating Cytotoxic T Lymphocytes

*Xiaohong Cen, Gengzhen Zhu, Junjie Yang, Jianjun Yang, Jiayin Guo, Jiabing Jin, Kutty Selva Nandakumar, Wei Yang, Hang Yin, Shuwen Liu, and Kui Cheng**

Supporting Information

TLR1/2 Specific Small-molecule Agonist Suppresses Leukemia cancer cell growth by stimulating Cytotoxic T lymphocytes

*Xiaohong Cen†, Gengzhen Zhu†, Junjie Yang, Jianjun Yang, Jiayin Guo, Jiabing Jin, Kutty Selva Nandakumar, Wei Yang, Hang Yin, Shuwen Liu and Kui Cheng**

Note S1. HTS and SAR studies.

Figure S1. The NF-κB activation of SMU-Z1 can be inhibited by a NF-κB inhibitor, triptolide, in HEK Blue-hTLR2 cells.

Figure S2. SMU-Z1 dose-dependently actives the percentage of $CD4^{+}CFSE^{low}$ T cells and $CD8^{+}CFSE^{low}$ T cells for 48 h and 72 h.

Figure S3. Raw 264.7 and HEK Blue-hTLR2 were incubated with indicated concentration of SMU-Z1.

Figure S4. Comparison of RF04289, CU-T12-9 and compound 1 to 20 for SEAP gene expression.

Figure S5. Comparison of compound 23 to 44 for SEAP gene expression..

Figure S6. 2D NMR data of 28.

Table S1. Structure-activity relationship (SAR) studies of compounds 1-20 in activation of SEAP signaling in HEK-Blue hTLR2 cells.

Table S2. SAR studies of compounds 21-48 in activating SEAP signaling in HEK-Blue hTLR2 cells.

Scheme S1. Synthesis Routes of Compound 1-20.

Scheme S2. Synthesis Routes of Compound 21-28.

Scheme S3. Synthesis Routes of Compound 29-48.

Note S2. Synthesis, ^{1}H NMR, ^{13}C NMR and Mass spectrometry data of all the compounds.

Note S1. HTS and SAR studies

The HTS was carried out at the HTS core facility of the University of Colorado at Boulder. Briefly, HEK hTLR2 cells were seeded in duplicate at 20,000 cells/well in a total volume of 40 μL/well in 384-well plates. Cells were stimulated with the library compounds (10 μM) or Pam3CSK4 (100 ng/mL). After 24 hours incubation at 37 ^{o}C with 5% CO_2, 40 μL QUANTI-Blue (InvivoGen) reagent was added and readout the SEAP signaling at OD620.

Based on the results of high-throughput screening, RF04289 exhibits characteristics superior to those of other parent structures, which possess a structure of thiazole bridged to trifluorophenyl. Coincidentally, our previous studies also found that similar imidazole bridged trifluoromethyl structures (CU-T12-9) also have very good TLR2 activation. With these in mind, we first changed imidazole to thiazole to observe the spinning effect (Scheme S1). Hydroxyl group at the meta position of CF_3 and an imidazole as the intermediate bridging group also studied for their activity (Scheme S2). The potency of all the compounds was detected in HEK-Blue hTLR2 cells by monitoring the secreted alkaline phosphatase (SEAP) reporter activation with untreated cell samples as the baseline control.

In the beginning, compounds **1** to **10** were chosen to detect the preference of thiazole over imidazole of compound RF04289 (Scheme S1). Compounds **11** to **20** were chosen to compare with compounds **1** to **10** for exploring the functions of hydroxyl group. The EC_{50} of the compounds **11** to **20** were decreased compared to compounds, **1** to **10,** on the whole, which demonstrated the vital role of hydroxyl group for activity holding (Fig. S4). Next, we compared the compounds, **11** to **20**, with **1** to **10**, and identified imidazole as the more preferable group compared to thiazole (Table S1).

On the basis of above SAR analysis, it would appear that the hydroxyl group at the meta position of CF_3 and an imidazole as the intermediate bridging group bring favor to functional activity. Then we developed the compounds, **23** to **26** (Scheme S2), to investigate the small structural variations of these compounds on their biological activity effects, and the preliminary results indicated that a combination of electronic and hydrolytic properties play a role in improving the biological activity of the synthesized compounds. In the analogous, compounds, **25-26**, with methyl formate group, formyl group were introduced instead of the nitro group in the compound **23** at R6 position. The activity of compound **26** was reduced 10-fold compared to that of compound, **23**. The amide bond in compound **26**, which is replaced by an ester linkage (**25**), shows a further 10-fold decrease in activity, indicating that the non-hydrolyzable group is very important for its activity (Table S2 and Fig. S5). As a result, we finally identified that 2-(1-(2-(methylamino)-5-nitrophenyl)-1*H*-imidazol-4-yl)-5-(trifluoromethyl) phenol (compound **23**, SMU-Z1) has significantly improved activity (about 10 fold) in TLR2 activation (EC_{50} = 4.88±0.79 nM) compared to the parent compound RF04289. Compounds **27** and **28** were synthesized by one-step unexpected reaction of the substrate with thionyl chloride, which have never been reported by others that directly introduce a chloride on the benzene ring under the thionyl chloride condition. Compound **28** was further confirmed using 2D-NMR for the incorporation of chloride ion (Fig. S6).

The effect of substitution on hydroxyl group attached to the aromatic ring was also studied. Methylation or ethylation of the hydroxyl group gave a significant decrease (20 to 30000 folds) of the TLR2 activity (Table S2 and Fig. S2). Notably, the activity was significantly decreased after benzylation (SMU-Z1 vs compound, **31**), which indicated that the short steric effect plays a dominant effect to the activity. Switching the hydroxyl with an ether bond

significantly alters the activity (compounds, **32**-**35**, Table S2 and Scheme S3). However, replacing the ether bond by ester bond regenerated some of the TLR2 activities (compounds, **30** versus **36**), and with the extension of the alkyl or aryl carbon chain showed a downward trend, but did not completely abrogated the activity (compounds, **36**-**40**, Table S2 and Fig. S5). These results imply that the ester group could potentially be hydrolyzed to hydroxyl groups in the cellular environments. When a carboxylic group (compound, **40**) or sulfonic acid group (compound, **41**) was incorporated into the structure, the TLR1 and TLR2 activity was not significantly affected. To verify whether the SMU-Z1 potential binding to TLR1 and TLR2, a series of biotinylated compounds were designed and synthesized having different linking groups with different lengths. Among them, compound **45** keeps the best TLR1 and TLR2 activation effect with an EC_{50} at 764.48±10.18 nM and the activity was found to be gradually lost with prolonging of the linker (Table S2). These data indicates that minor modifications on the best-in-class structure of SMU-Z1 had a profound effect on its agonist activity. Extensive SAR analysis thus led us to optimize a novel small-molecule agonist to TLR2, SMU-Z1, and hence we further evaluated its potency in the following experiments.

All of the solvents and reagents used were obtained commercially and used as received. The solvents were removed under reduced pressure using standard rotary evaporators. Compounds were purified using flash chromatography (FC) (Silica gel 60 or alumina-B, 200-400 mesh, Sorbent Tech.) or recrystallization. Purity for all final compounds was confirmed to be greater than 97% purity by ^{1}H NMR, ^{1}H NMR spectra were recorded at 400 MHz in $CDCl_3$ or $(CD_3)_2SO$ using residual solvent peaks ($CDCl_3$, δ 7.28; $(CD_3)_2SO$, δ 2.51) as the internal standard. ^{13}C NMR spectra were recorded at 101 MHz in $CDCl_3$ or $(CD_3)_2SO$ using residual $CHCl_3$ (77.16 ppm) and $(CD_3)_2SO$ (39.52 ppm) as the internal reference.

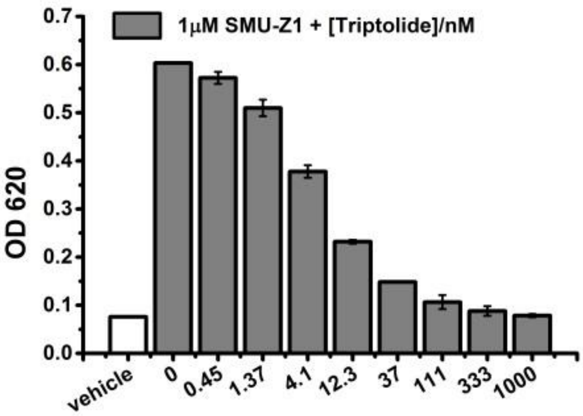

Figure S1. The NF-κB activation of SMU-Z1 can be inhibited by a NF-κB inhibitor, triptolide, in HEK Blue-hTLR2 cells.

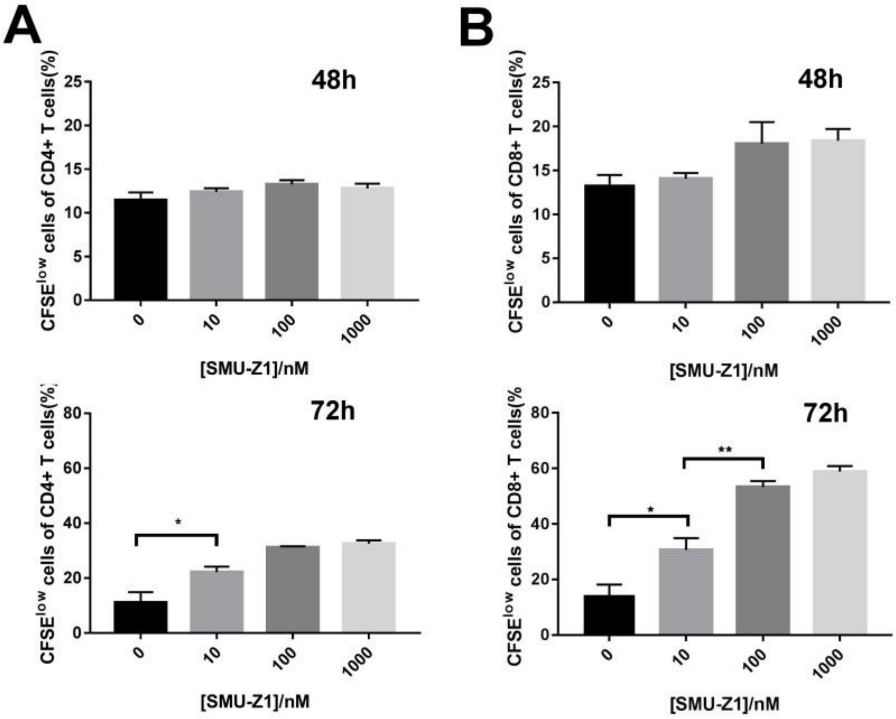

Figure S2. SMU-Z1 dose-dependently actives the percentage of $CD4^{+}CFSE^{low}$ T cells and $CD8^{+}CFSE^{low}$ T cells for 48 h and 72 h. Data are means ± SD of triplicates and representative of three independent experiments. *p<0.01, **p<0.001.

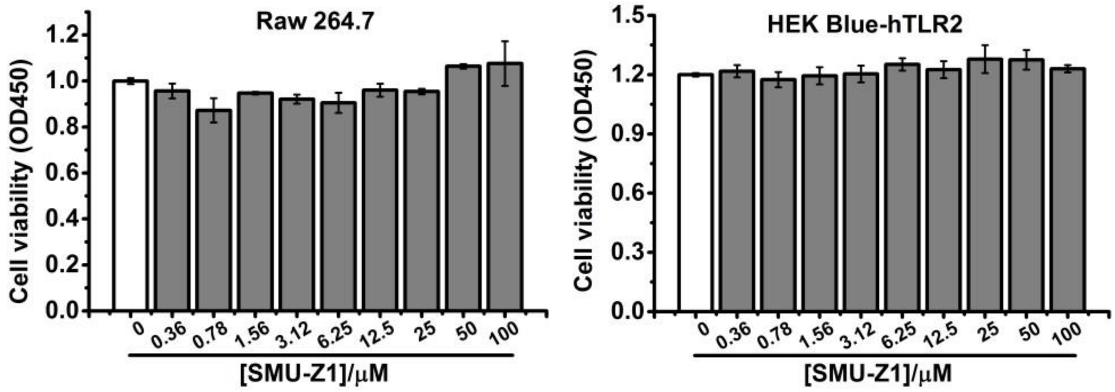

Figure S3. Raw 264.7 and HEK Blue-hTLR2 were incubated with indicated concentration of SMU-Z1. The cell viability was assessed with the CCK-8 kit (Bimake, B34304). Cells have no toxicity issues as concentration of SMU-Z1 up to 100 μM.

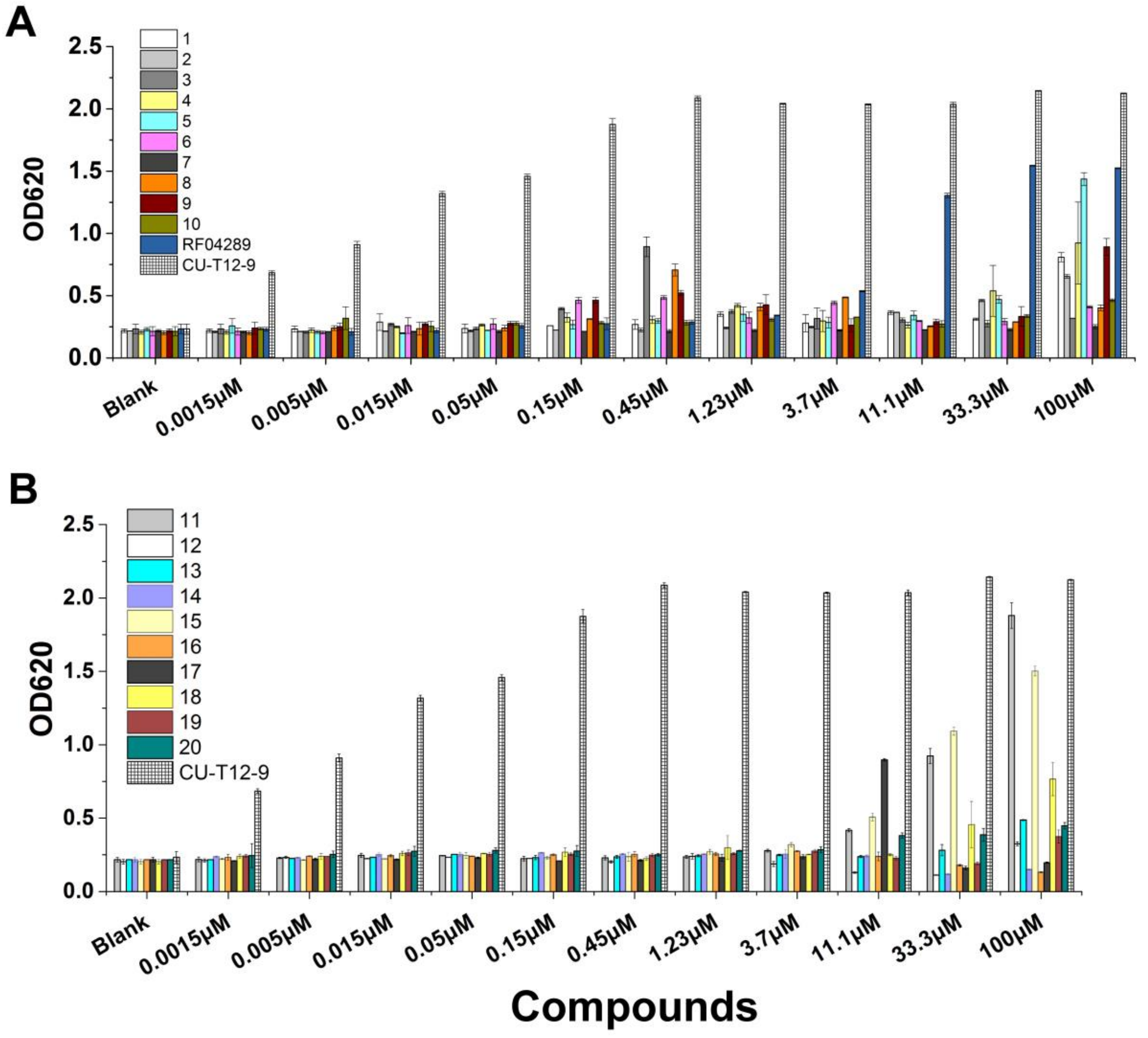

Figure S4. Comparison of RF04289, CU-T12-9 and compound 1 to 20 for SEAP gene expression. HEK-Blue hTLR2 cells were incubated with 1 to 20, RF04289 or CU-T12-9 for 24 hours, and the activation was measured by Quati-Blue reagent (Invivogen) to detect the secreted embryonic alkaline phosphatase (SEAP) signalling in the culture supernatants at OD620. Data presented are mean ± SD and the figures shown are representative of three independent experiments.

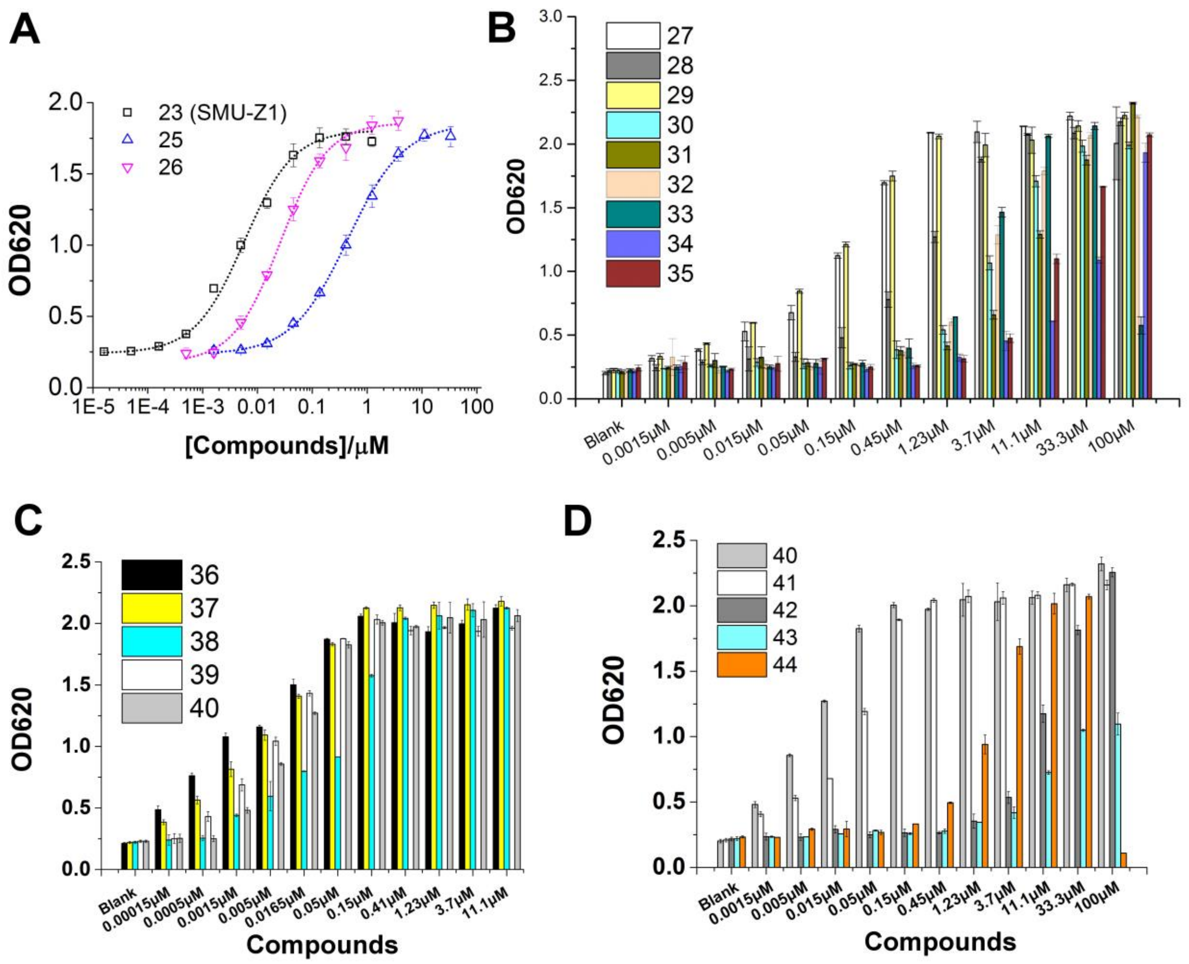

Figure S5. Comparison of compound 23 to 44 for SEAP gene expression. HEK-Blue hTLR2 cells were incubated with the indicated concentrations of 23 to 44 for 24 hours, and the activation was measured by Quati-Blue reagent (Invivogen) to detect the secreted embryonic alkaline phosphatase (SEAP) signalling in the culture supernatants at OD620. Data presented are mean ± SD and the figures shown are representative of three independent experiments.

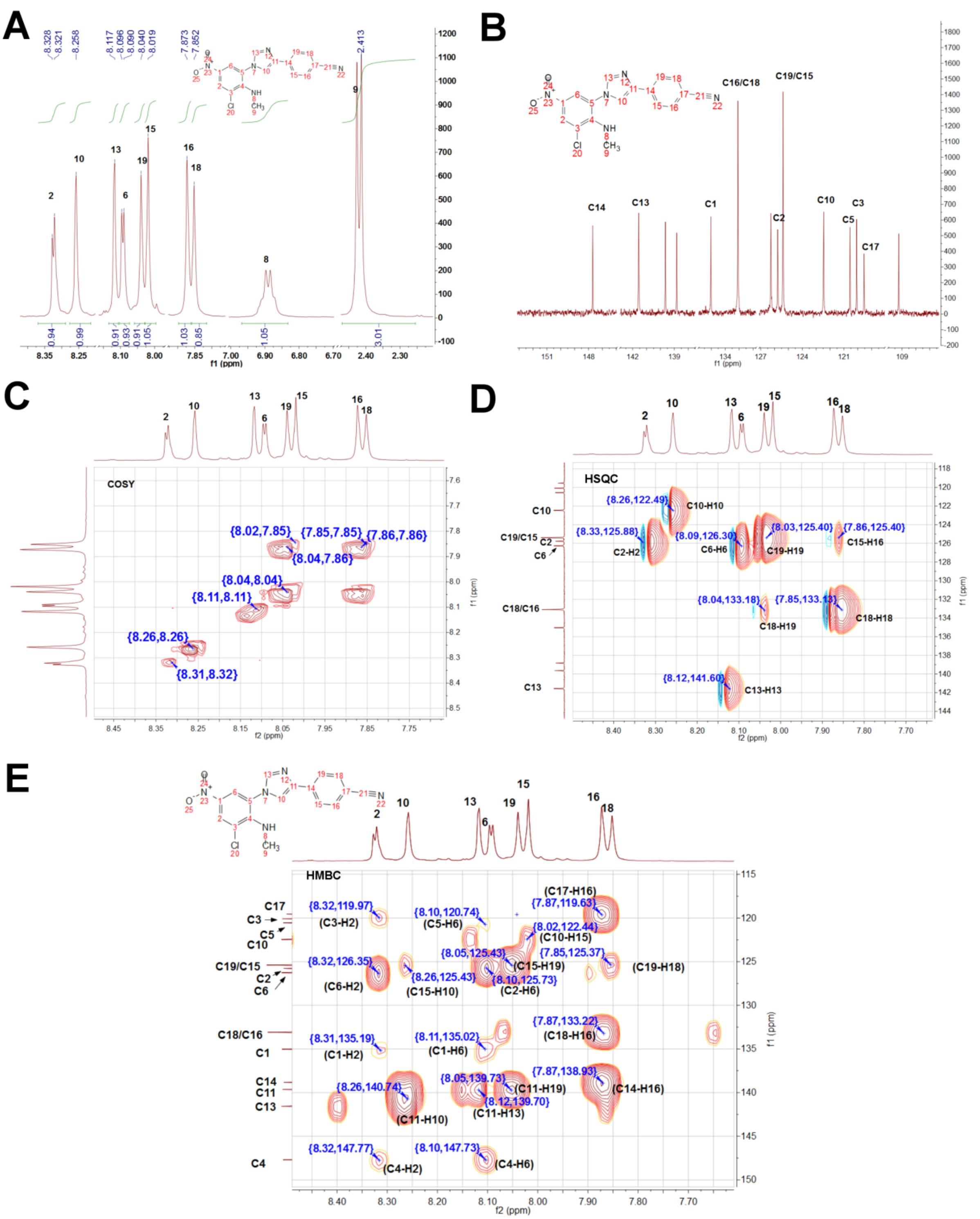

Figure S6. 2D NMR data of 28: (A) ^{1}H NMR, (B) ^{13}C NMR, (C) COSY, (D) HSQC and (E) HMBC.

Table S1. Structure-activity relationship (SAR) studies of compounds 1-20 in activation of SEAP signaling in HEK-Blue hTLR2 cells.

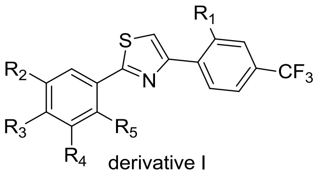

derivative I

Compound	R_1	R_2	R_3	R_4	R_5	EC_{50} (nM)
1	H	NO_2	H	H	Cl	>100000[a]
2	H	NO_2	H	H	$NHCH_3$	>100000
3	H	H	H	H	H	NA
4	H	CF_3	H	H	H	>100000[a]
5	H	H	CF_3	H	H	>100000
6	H	H	H	H	CF_3	NA[b]
7	H	CF_3	H	CF_3	H	NA[b]
8	H	H	OCH_3	H	H	NA[b]
9	H	H	NO_2	H	H	>100000[a]
10	H	CH_3	H	H	H	NA[b]
11	OH	NO_2	H	H	Cl	>100000[a]
12	OH	NO_2	H	H	$NHCH_3$	NA[b]
13	OH	H	H	H	H	NA[b]
14	OH	CF_3	H	H	H	NA[b]
15	OH	H	CF_3	H	H	>100000
16	OH	H	H	H	CF_3	NA[b]
17	OH	CF_3	H	CF_3	H	NA[b]
18	OH	H	OCH_3	H	H	>100000[a]
19	OH	H	NO_2	H	H	NA[b]
20	OH	CH_3	H	H	H	NA[b]
RF04289	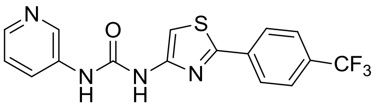					6280.05±487.74

[a] The highest SEAP activation observed is at least 50% lower than the highest activation of SMU-Z1. [b] No activity was detected at the tested concentrations up to 100 µM. NA means no activating effect. The numbers of EC_{50} are determined from at least three independent repeats.

Table S2. SAR studies of compounds 21-48 in activating SEAP signaling in HEK-Blue hTLR2 cells.

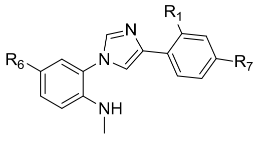

21-26, 29-48

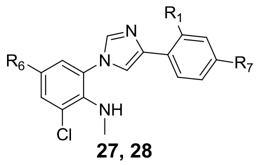

27, 28

Compound	R_1	R_6	R_7	EC_{50} (nM)
21	H	NO_2	CN	92.97±13.03
22	H	NO_2	OCF_3	24.87±6.15
23(SMU-Z1)	OH	NO_2	CF_3	4.88±0.79
25	OH	$COOCH_3$	CF_3	432.86±0.05
26	OH	$CONHCH_3$	CF_3	45.57±8.15
27	OH	NO_2	CF_3	192.19±17.19
28	H	NO_2	CN	985.72±79.49
29	OCH_3	NO_2	CF_3	142.42±25.13
30	OCH_2CH_3	NO_2	CF_3	3845.23±340.89
31	$OCH_2C_6H_5$	NO_2	CF_3	15360.57±1899.29
32	OCH_2CH_2Br	NO_2	CF_3	4074.84±227.88
33	$OCH_2CH_2CH_2Br$	NO_2	CF_3	2853.19±407.35
34	$O(CH_2)_2O(CH_2)_2Br$	NO_2	CF_3	>100000[a]
35	$O(CH_2)_2O(CH_2)_2OH$	NO_2	CF_3	16955.54±1930.15
36	$OCOCH_3$	NO_2	CF_3	8.83±3.65
37	$OCO(CH_2)_6CH_3$	NO_2	CF_3	12.32±2.31
38	$OCO(CH_2)_{10}CH_3$	NO_2	CF_3	90.69±4.69
39	$OCOC_6H_5$	NO_2	CF_3	7.65±2.79
40	$OCOCH=CHC_6H_5$	NO_2	CF_3	10.66±1.58
41	OSO_3H	NO_2	CF_3	49.13±3.82
42	$O(CH_2)_2O(CH_2)_2O(CH)_2I$	NO_2	CF_3	15694.56±1229.11
43	OCH_2CONH_2	NO_2	CF_3	>100000 [a]
44	$O(CH_2)_2NH_2$	NO_2	CF_3	2122.13±641.25
45	$O(CH_2)_2$NH-Biotin	NO_2	CF_3	764.48±10.18
46	$O(CH_2)_2$O-Biotin	NO_2	CF_3	10089.23±588.15
47	$O(CH_2)_2O(CH_2)_2$O-Biotin	NO_2	CF_3	13566.79±423.50
48	$O(CH_2)_2O(CH_2)_2O(CH_2)_2$O-Biotin	NO_2	CF_3	13582.14±414.31

[a] The highest SEAP activation observed is at least 50% lower than the highest activation of SMU-Z1. NA means no activating effect. The numbers of EC_{50} are determined from at least three independent repeats

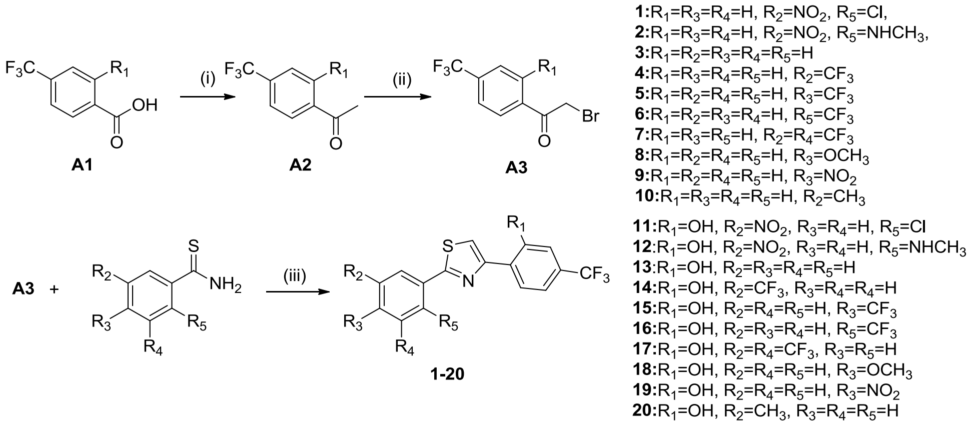

Scheme S1. Synthesis Routes of Compound 1-20. Reagents and conditions: (i) CH_3Li, THF, 0 ^oC to rt, 3 h; (ii) tetrabutylammonium tribromide, DCM, MeOH, rt, 10 h; (iii) EtOH, rf, 12 h.

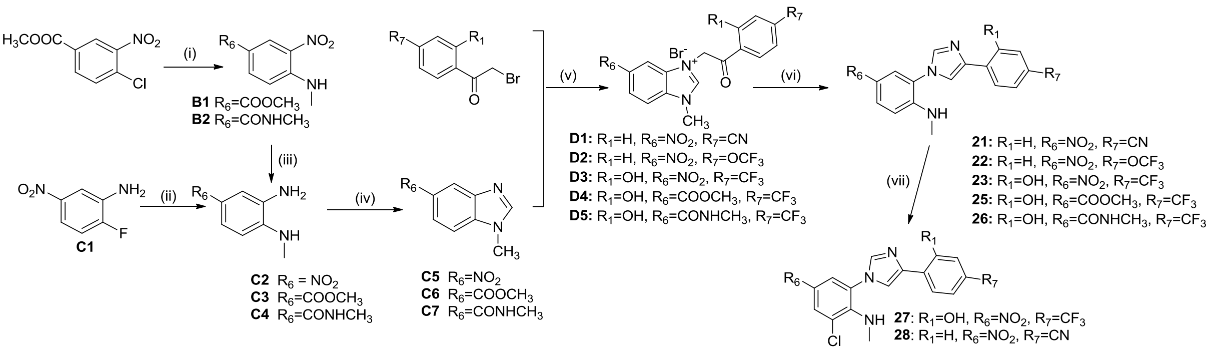

Scheme S2. Synthesis Routes of Compound 21-28. Reagents and conditions: (i) $CH_3NH_2{\cdot}HCl$, NaOH, EtOH, rf, 24 h; B5: $CH_3NH_2{\cdot}H_2O$(w=40%), rt, 1 h: (ii) $CH_3NH_2{\cdot}H_2O$(w=40%), rt, 12 h; (iii) Zn, AcOH, EtOH, 0 ^oC to rt, 3 h; (iv) $HC(OC_2H_5)_3$, con HCl, DMF, rt, 3 h; (v) EtOH, rf, 12 h; (vi) CH_3COOH, $AcONH_4$, rf, 12 h (vii) $SOCl_2$, 0 ^oC to rt, 24 h.

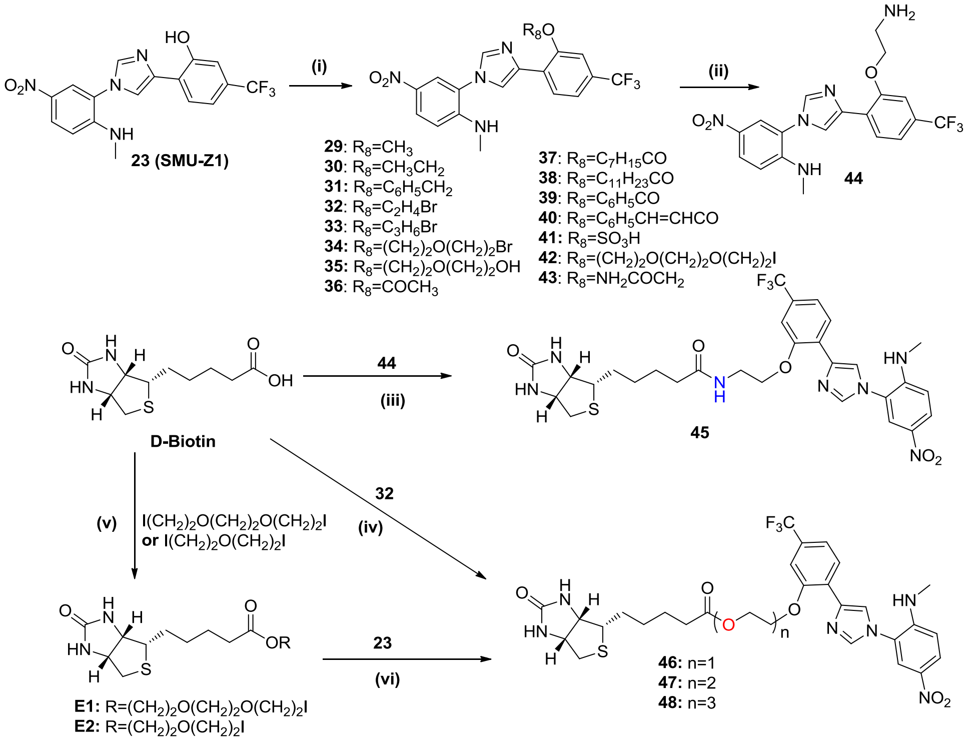

Scheme S3. Synthesis Routes of Compound 29-48. Reagents and conditions: (i) **29**: CH_3I, acetone, K_2CO_3, rf, 12 h; **30**: CH_3CH_2I, acetone, K_2CO_3, rf, 12 h; **31**: $C_6H_5CH_2Br$, acetone, K_2CO_3, rf,12 h; **32**:1,2-dibromoethane, acetone, K_2CO_3, rf, 12 h; **33**: 1,3-dibromopropane, acetone, K_2CO_3, rf, 12 h; **34**: $Br(CH_2)_2O(CH_2)_2Br$, acetone, K_2CO_3, rf, 12 h; **35**: $Cl(CH_2)_2O(CH_2)_2OH$, acetone, K_2CO_3, rf, 12 h; **36**: CH_3COCl, Et_3N, DCM, 0 ^oC, 1 h; **37**: $C_7H_{15}COCl$, Et_3N, DCM, 0 ^oC, 1 h; **38**:$C_{11}H_{23}COCl$, Et_3N, DCM, 0 ^oC, 1 h; **39**:C_6H_5COCl, Et_3N, DCM, 0 ^oC, 1 h; **40**:$C_6H_5CH=CHCOCl$, Et_3N, DCM, 0 ^oC, 1 h; **41**: SO_3/Pyridine, Et_3N, THF, rf, 6 h; **42**: $I(CH_2)_2O(CH_2)_2O(CH_2)_2I$, acetone, K_2CO_3, rf, 12 h; **43**: NH_2COCH_2Cl, 2-Butanone, KI, rf, 12 h; (ii) **32**, $NH_3{\cdot}H_2O$, KI, THF, 12 h; (iii) HATU, DIPEA, rt, 12 h. (iv) NaH, KI, DMSO, rt, 12 h; (v) NaH, DMSO, rt, 12 h; (vi) K_2CO_3, acetone, rf, 12 h.

Note S2. Structure Characterization

^{1}H NMR, ^{13}C NMR and Mass spectrometry of all the compounds.

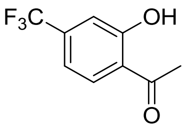

*1-(2-hydroxy-4-(trifluoromethyl)phenyl)ethanone (**A2**).* To a solution of 2-hydroxy-4-(trifluoromethyl) benzoic acid (1.0 g, 4.85 mmol) in tetrahydrofuran (10 mL) in the temperature of under 5 °C was added methyllithium (10 mL) inside (slowly add, in case of producing CH_4). The reaction mixture was allowed to room temperature for 3 hours and monitored by TLC (Scheme S1). Then the crude mixture was stopped by water (20 mL) and extracted sequentially with ethyl acetate (3 × 50 mL). The organic layer was then dried over Na_2SO_4, and concentrated under reduced pressure. The crude product was purified by flash column chromatography on silica gel (petroleum ether(PE):ethyl acetate(EA)=8:1) to provide the product as yellow oil (750 mg, 75.8 %). ^{1}H NMR (400 MHz, $CDCl_3$) δ 12.30 (s, 1H), 7.86 (d, J = 8.3 Hz, 1H), 7.23 (s, 1H), 7.14 (d, J = 8.2 Hz, 1H), 2.71 – 2.66 (m, 3H). ^{13}C NMR (101 MHz, $CDCl_3$) δ 204.19, 162.11, 137.03, 131.31, 124.30, 121.48, 115.73, 115.18, 26.68. MS (ESI-TOF) for $C_9H_7F_3O_2$ $[M - H]^-$ calculated 203.1, found 203.2.

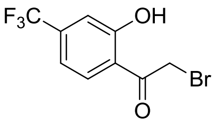

*2-bromo-1-(2-hydroxy-4-(trifluoromethyl)phenyl)ethanone (**A3**).* To a solution of 2- ethanone-5- trifluoromethyl-1-phenol (406 mg, 1.5 mmol) in DCM (10 mL) and MeOH (10mL), tetrabutylammonium (723 mg, 1.5 mmol) was resolved in DCM (10 mL) and was added into the former mixture by dropwise within 30 minutes. Once the color became yellow, the reaction finished. The crude product was purified by flash column chromatography on silica gel (PE:EA=8:1) to provide the product as yellow oil (750 mg, 75.8 %). ^{1}H NMR (400 MHz, $CDCl_3$) δ 11.74 (s, 1H), 7.90 (d, J = 8.4 Hz, 1H), 7.28 (s, 1H), 7.18 (d, J = 7.3 Hz, 1H), 4.47 (s, 2H). ^{13}C NMR (101 MHz, $CDCl_3$) δ 190.35, 136.56, 134.92, 129.27, 125.89, 124.70, 121.99, 30.27. MS (ESI-TOF) for $C_9H_6BrF_3O_2$ $[M - H]^-$ calculated 282.0, found 282.2.

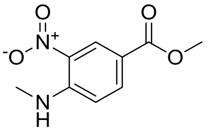

*methyl 4-(methylamino)-3-nitrobenzoate (**B1**).*

To a mixture of methyl 4-chloro-3-nitrobenzoate (215.59 mg, 1 mmol), methylamine hydrochloride (335 mg, 5 mmol) and sodium hydroxide (200 mg, 5 mmol) in EtOH (10mL), the mixture was reacted in reflux temperature for 24 hours (Scheme S2). The crude product was purified by flash column chromatography on silica gel (PE:EA=8:1) to provide the product as yellow solid (873.5 mg, 96.4 %). m.p. 177.5-179.1 °C. ^{1}H NMR (400 MHz, $CDCl_3$) δ 8.80 (d, J = 2.2 Hz, 1H), 8.47 (d, J = 2.0 Hz, 1H), 7.96 (dd, J = 9.1, 1.8 Hz, 1H), 7.00 (d, J = 9.1 Hz, 1H), 3.17 – 3.12 (m, 3H), 3.08 (s, 3H). ^{13}C NMR (101 MHz, $CDCl_3$) δ 148.56, 133.67, 127.98, 126.71, 114.21, 44.72, 30.00. MS (ESI-TOF) for $C_9H_{10}N_2O_4$ $[M + H]^+$ calculated 211.2, found 211.0.

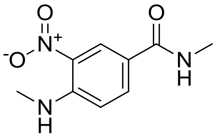

N-methyl-4-(methylamino)-3-nitrobenzamide ***(B2)***. Mixed methyl 4-chloro-3-nitrobenzoate (431.2 mg, 2 mmol) and methylamine solution (40 % in H_2O, 10mL) in the 25 ml round-bottomed flask, and the mixture was reacted in room temperature for 1 hour. The crude product was purified by flash column chromatography on silica gel (PE:EA=8:1) to provide the product as yellow solid (382.8 mg, 91.5 %). m.p. 187.2-189.5 °C. ^{1}H NMR (400 MHz, DMSO) δ 8.64 (s, 1H), 8.53 – 8.29 (m, 2H), 8.01 (d, *J* = 9.1 Hz, 1H), 7.05 (d, *J* = 9.0 Hz, 1H), 3.01 (d, *J* = 4.9 Hz, 3H), 2.78 (d, *J* = 4.4 Hz, 3H). ^{13}C NMR (101 MHz, DMSO) δ 165.17, 147.46, 134.92, 130.67, 126.11, 121.09, 114.48, 30.23, 26.61.^{13}C NMR (101 MHz, DMSO) δ 167.87, 140.14, 134.49, 122.82, 117.73, 113.29, 107.81, 30.33, 26.56. MS (ESI-TOF) for $C_9H_{11}N_3O_3$ $[M + H]^+$ calculated 210.2, found 210.2.

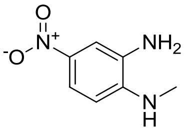

N^1-methyl-4-nitrobenzene-1,2-diamine ***(C2)***. To a mixture of 2-fluoro-5-nitroaniline (20 g, 128.11 mmol) and methylamine solution (concentration 40 %) (20 mL) was reacted for 48 hours. Then the mixture was poured into water to afford the crude solid and then purified by flash column chromatography on silica gel (PE:EA=4:1) to provide the product as red solid (24.3 g, 90.2 %). m.p. 149.3-151.9 °C. ^{1}H NMR (400 MHz, DMSO) δ 7.56 (dd, *J* = 8.8, 2.7 Hz, 1H), 7.41 (d, *J* = 2.7 Hz, 1H), 6.43 (d, *J* = 8.9 Hz, 1H), 6.12 (d, *J* = 4.7 Hz, 1H), 5.09 (s, 2H), 2.85 (d, *J* = 4.8 Hz, 3H). ^{13}C NMR (101 MHz, DMSO) δ 144.06, 136.95, 134.83, 116.44, 107.40, 106.90, 30.07. MS (ESI-TOF) for $C_7H_9N_3O_2$ $[M + H]^+$ calculated 168.2, found 168.1.

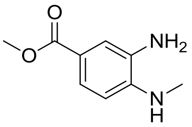

methyl 3-amino-4-(methylamino)benzoate ***(C3)***. To a mixture of methyl 4-(methylamino)-3-nitrobenzoate (315.3 mg, 1.5 mmol) in EtOH (10mL) with zinc powder (560 mg, 10 mmol), the mixture was added by acetic acid (5 mL) and reacted in room temperature for 3 hours. The crude product was purified by flash column chromatography on silica gel (PE:EA=1:1) to provide the product as gray solid (113.79 mg, 42.1 %). m.p. 156.3-158.1 °C. ^{1}H NMR (400 MHz, $CDCl_3$) δ 7.64 (dd, *J* = 8.3, 1.9 Hz, 1H), 7.42 (d, *J* = 1.9 Hz, 1H), 6.61 (d, *J* = 8.3 Hz, 1H), 3.87 (s, 3H), 3.52 (s, 2H), 2.93 (s, 3H). ^{13}C NMR (101 MHz, $CDCl_3$) δ 167.55, 144.01, 132.17, 124.34, 118.81, 117.88, 108.82, 51.56, 30.44. MS (ESI-TOF) for $C_8H_7N_3O_2$ $[M + H]^+$ calculated 181.2, found 181.2.

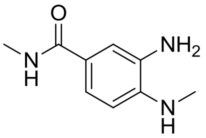

3-amino-N-methyl-4-(methylamino)benzamide ***(C4)***. To a mixture of N-methyl-4-(methylamino)-3-nitrobenzamide (418.4 mg, 2 mmol) in EtOH (10mL) with zinc powder (560 mg, 10 mmol), the mixture was added by acetic acid (5 mL) and reacted in room temperature for 3 hours. The crude product was purified by flash column chromatography on silica gel (PE:EA=1:1) to provide the product as gray solid (117.74 mg, 43.8 %). m.p. 159.8-160.1 °C. ^{1}H NMR (400 MHz, DMSO) δ 7.90 (d, *J* = 4.4 Hz, 1H), 7.25 – 6.99 (m, 2H), 6.37 (d, *J* = 7.9

Hz, 1H), 5.09 (d, J = 4.9 Hz, 1H), 4.58 (s, 2H), 2.75 (dd, J = 11.0, 4.7 Hz, 6H). ^{13}C NMR (101 MHz, DMSO) δ 167.87, 140.14, 134.49, 122.82, 117.73, 113.29, 107.81, 30.33, 26.56. m.p. 159.8-160.1 °C. MS (ESI-TOF) for $C_9H_{13}N_3O$ $[M + H]^+$ calculated 180.2, found 180.2.

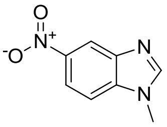

*1-methyl-5-nitro-1H benzo[d]imidazole (**C5**).* To a solution of DMF (4 mL) containing N1-methyl-4-nitrobenzene-1,2-diamine (251 mg,1.5 mmol) and triethoxymethane (10 mL) was added the concentrated hydrochloric acid (12 N solution, 167 µL, 5 mmol). The mixture solution was stirred at room temperature for 2 hours. The reaction was monitored by TLC. After reaction the mixture was filtered and purified by flash column chromatography on silica gel (EA: MeOH:Et_3N=95:3:2) as eluent to provide 1-methyl-5-nitro-1H-benzo[d]imidazole as light yellow solid (90 mg, 33.7 %). m.p. 209.2-210.7 °C. ^{1}H NMR (400 MHz, DMSO) δ 8.56 (d, J = 2.2 Hz, 1H), 8.51 (s, 1H), 8.21 (dd, J = 8.9, 2.2 Hz, 1H), 7.83 (d, J = 8.9 Hz, 1H), 3.94 (s, 3H). ^{13}C NMR (101 MHz, DMSO) δ 149.22, 143.13, 142.85, 139.41, 118.31, 115.97, 111.43, 31.66. MS (ESI-TOF) for $C_8H_7N_3O_2$ $[M + H]^+$ calculated 178.2, found 178.2.

Compounds C6 and C7 were prepared as the similar synthesis route for C5.

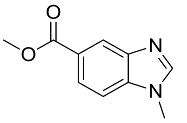

*methyl 1-methyl-1H-benzo[d]imidazole-5-carboxylate (**C6**).* White solid (212 mg, 68 %). m.p. 230.5-231.3 °C. ^{1}H NMR (400 MHz, DMSO) δ 8.35 (s, 1H), 8.26 (s, 1H), 7.91 (d, J = 8.5 Hz, 1H), 7.69 (d, J = 7.7 Hz, 1H), 3.88 (d, 6H). ^{13}C NMR (101 MHz, DMSO) δ 167.20, 147.24, 143.32, 138.34, 123.70, 121.45, 110.83, 52.38, 31.34. MS (ESI-TOF) for $C_{10}H_{10}N_2O_2$ $[M + H]^+$ calculated 191.2, found 191.1.

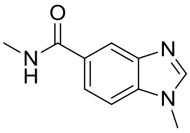

*N,1-dimethyl-1H-benzo[d]imidazole-5-carboxamide (**C7**).* Gray solid (367 mg, 78 %). m.p. 237.5-238.5 °C. ^{1}H NMR (400 MHz, DMSO) δ 8.42 (d, J = 4.3 Hz, 1H), 8.28 (s, 1H), 8.20 (d, J = 0.8 Hz, 1H), 7.82 (dd, J = 8.5, 1.4 Hz, 1H), 7.62 (d, J = 8.5 Hz, 1H), 3.87 (s, 3H), 2.82 (d, 3H). ^{13}C NMR (101 MHz, DMSO) δ 167.54, 146.48, 143.28, 136.84, 128.67, 122.21, 118.84, 110.22, 31.21, 26.77. MS (ESI-TOF) for $C_{10}H_{11}N_3O$ $[M + H]^+$ calculated 190.2, found 190.2.

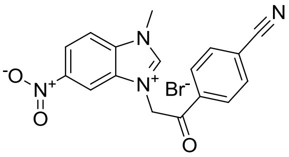

*3-(2-(4-cyanophenyl)-2-oxoethyl)-1-methyl-5-nitro-1H-benzo[d]imidazol-3-ium bromide (**D1**).* To a solution of 1-methyl-5-nitro-1H benzo[d]imidazole (177 mg, 1 mmol) and 4-(2-bromoacetyl)benzonitrile (224 mg, 1 mmol) in EtOH (10 mL) was refluxed under nitrogen atmosphere for 12h. The reaction was monitored by TLC. The solvent was concentrated under reduced pressure to give the crude product. And acetone (20 mL) was added inside under ultraphonic to provide the white solid. Finally the white solid was recrystallized in MeOH (5 mL) to give the pure white solid (366 mg, 91.4 %). m.p. 244.0-244.9 °C. ^{1}H NMR (400 MHz,

DMSO) δ 9.97 (d, J = 11.9 Hz, 1H), 9.30 (d, J = 2.0 Hz, 1H), 8.58 (dd, J = 9.2, 1.8 Hz, 1H), 8.36 (d, J = 9.2 Hz, 1H), 8.29 (d, J = 8.5 Hz, 2H), 8.19 (d, J = 8.5 Hz, 2H), 6.57 (d, J = 5.3 Hz, 2H), 4.28 (s, 3H). ^{13}C NMR (101 MHz, DMSO) δ 190.92, 148.16, 146.34, 137.42, 135.54, 133.38, 132.03, 129.53, 122.05, 118.44, 116.48, 115.71, 111.87, 54.85, 34.78. MS (ESI-TOF) for $C_{17}H_{13}BrN_4O_3$ [M + H]$^+$ calculated 401.0244, found 401.0241.

Compounds D2 and D6 were prepared as the similar synthesis route for B5.

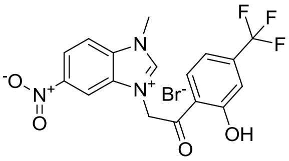

*3-(2-(2-hydroxy-4-(trifluoromethyl)phenyl)-2-oxoethyl)-1-methyl-5-nitro-1H-benzo[d]imidazol-3-ium bromide (**D3**).* White solid (411 mg, 89.4 %). m.p. 188.4-185.9 °C. ^{1}H NMR (400 MHz, DMSO) δ 11.92 (s, 1H), 9.98 (d, J = 10.7 Hz, 1H), 9.27 (d, J = 2.0 Hz, 1H), 8.56 (dd, J = 9.2, 1.7 Hz, 1H), 8.35 (d, J = 9.2 Hz, 1H), 8.03 (d, J = 8.2 Hz, 1H), 7.52 (d, J = 4.9 Hz, 1H), 7.34 (d, J = 8.3 Hz, 1H), 6.34 (s, 2H), 4.27 (s, 3H). ^{13}C NMR (101 MHz, DMSO) δ 190.98, 159.73, 148.31, 146.30, 135.51, 135.29, 132.08, 125.14, 124.76, 122.43, 121.88, 115.89, 115.52, 114.70, 112.03, 57.73, 34.62. MS (ESI-TOF) for $C_{17}H_{13}BrF_3N_3O_4$ [M + H]$^-$ calculated 460.0144, found 460.0147.

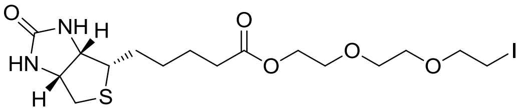

*2-(2-(2-iodoethoxy)ethoxy)ethyl 5-((3aS,4S,6aR)-2-oxohexahydro-1H-thieno[3,4-d]imidazol-4-yl) pentanoate (**E1**).* To a stired mixture of D-Biotin (244 mg, 1 mmol) in anhydrous DMSO (5 mL) and under nitrogen atmosphere was added sodium hydride (72 mg, 3 mmol) inside. Then adding 1,2-Bis(2-iodoethoxy) ethane(369 mg, 1 mmol) dropwise inside the flask (Scheme S3). The mixture was reacted at room temperature for 24 hours and was monitored by TLC. Then the crude mixture was stopped by water (20 mL) and extracted sequentially with ethyl acetate (3 × 50 mL) and half-saturate NH_4Cl solution. The organic layer was collected and dried over Na_2SO_4. The organic solution was again filtered and concentrated to dryness. The crude mixture was purified by column chromatography to afford the product as yellow solid (345.9 mg, 71%). m.p. 120.6-121.8 °C. ^{1}H NMR (400 MHz, $CDCl_3$) δ 5.75 (s, 1H), 5.21 (s, 1H), 4.61 – 4.47 (m, 1H), 4.40 – 4.17 (m, 3H), 3.84 – 3.63 (m, 8H), 3.29 (t, 2H), 3.18 (dd, 1H), 2.94 (dd, 1H), 2.76 (d, 1H), 2.40 (t, 2H), 1.78 – 1.61 (m, 5H), 1.48 (d, 2H). ^{13}C NMR (101 MHz, $CDCl_3$) δ 173.65, 163.80, 71.89, 70.43, 70.13, 69.17, 63.35, 61.90, 60.08, 55.53, 40.52, 33.74, 28.28, 28.18, 24.69, 2.96. MS (ESI-TOF) for $C_{16}H_{27}IN_2O_5S$ [M + H]$^+$ calculated 487.0758, found 487.0755.

Synthesis of compound E2 were carried out as reported for E1.

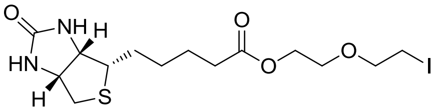

*2-(2-iodoethoxy)ethyl 5-((3aS,4S,6aR)-2-oxohexahydro-1H-thieno[3,4-d]imidazol-4-yl)pentanoate (**E2**).* White solid (508 mg, 73 %). m.p. 113.1-113.5 °C. ^{1}H NMR (400 MHz, $CDCl_3$) δ 5.95 (s, 1H), 5.40 (s, 1H), 4.60 – 4.45 (m, 1H), 4.40 – 4.29 (m, 1H), 4.26 (s, 2H), 3.75 (dt, 4H), 3.28 (t, 2H), 3.18 (d, 1H), 2.93 (dd, 1H), 2.77 (d, 1H), 2.40 (t, 2H), 1.84 – 1.57 (m, 4H), 1.48 (d, 2H). ^{13}C NMR (101 MHz, $CDCl_3$) δ 173.60, 163.96, 71.64, 68.61, 63.20, 61.92, 60.08, 55.55, 40.55, 33.76, 28.30, 24.69, 2.77. MS (ESI-TOF) for $C_{14}H_{23}IN_2O_4S$ [M + H]$^+$ calculated 443.0496, found 443.0495.

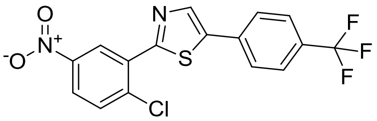

2-(2-chloro-5-nitrophenyl)-5-(4-(trifluoromethyl)phenyl)thiazole (***1***). To a mixture of 2-chloro-5-nitrobenzothioamide (243 mg, 1.12 mmol) and 2-bromo-1-(4-(trifluoromethyl)phenyl)ethanone (300 mg, 1.12 mmol) were resolved in anhydrous ethyl alcohol (5 mL) together. Then reacted for 4 hours in reflux temperature and monitored by TLC.[1] Once stopped, the reaction solvent was dried under reduced pressure and purified by flash column chromatography on silica gel (PE-EA=4:1) and recrystallized to provide the product as white solid (276 mg, 64.1 %). m.p. 169.2-170.2 °C. ^{1}H NMR (400 MHz, $CDCl_3$) δ 9.34 (d, J = 2.8 Hz, 1H), 8.23 (dd, J = 8.8, 2.8 Hz, 1H), 8.16 (d, J = 8.1 Hz, 2H), 7.86 (s, 1H), 7.74 (dd, J = 12.4, 8.5 Hz, 3H). ^{13}C NMR (101 MHz, $CDCl_3$) δ 99.97, 160.97, 153.95, 146.81, 137.96, 136.88, 132.89, 131.83, 130.50, 126.68, 125.84, 124.31, 117.21. MS (ESI-TOF) for $C_{16}H_8ClF_3N_2O_2S$ $[M + H]^+$ calculated 385.0020, found 385.0025.

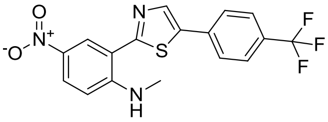

N-methyl-4-nitro-2-(5-(4-(trifluoromethyl)phenyl)thiazol-2-yl)aniline (***2***). To a mixture of 2-(2-chloro-5-nitrophenyl)-5-(4-(trifluoromethyl)phenyl)thiazole (300 mg, 0.78 mmol) and methylamine hydrochloride (263 mg, 3.9 mmol) were mixed together in EtOH (15 mL), then reacted in room temperature for 1 hour and was monitored by TLC. Then the organic mixture was collected and dried over Na_2SO_4. The organic solution was again concentrated to dryness. The crude mixture was purified by column chromatography (PE-EA=4:1) and recrystallized to afford the product as yellow solid (284.9 mg, 96.3 %). m.p. 145.3-145.9 °C. ^{1}H NMR (400 MHz, DMSO) δ 9.40 (d, J = 5.0 Hz, 1H), 8.57 – 8.37 (m, 2H), 8.24 (d, J = 8.2 Hz, 2H), 8.19 (dd, J = 9.3, 2.5 Hz, 1H), 7.85 (d, J = 8.3 Hz, 2H), 6.97 (d, J = 9.5 Hz, 1H), 3.14 (d, 3H). ^{13}C NMR (101 MHz, DMSO) δ 167.39, 152.95, 151.80, 137.26, 135.79, 127.21, 126.27, 125.45, 116.73, 113.68, 111.54, 30.35. MS (ESI-TOF) for $C_{17}H_{12}F_3N_3O_2S$ $[M + H]^+$ calculated 380.0675, found 386.0674.

Compounds 3 to 11 were prepared similar as 1.

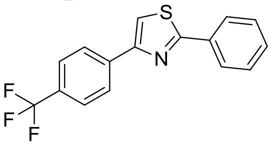

2-phenyl-4-(4-(trifluoromethyl)phenyl)thiazole (***3***). White solid (299.2 mg, 98.2%). m.p. 126.1-127.0 °C. ^{1}H NMR (400 MHz, $CDCl_3$) δ 8.14 (d, J = 8.2 Hz, 2H), 8.07 (dd, J = 7.4, 2.1 Hz, 2H), 7.73 (d, J = 8.3 Hz, 2H), 7.61 (s, 1H), 7.50 (dd, J = 5.0, 2.3 Hz, 3H). ^{13}C NMR (101 MHz, $CDCl_3$) δ 168.34, 154.65, 137.65, 133.41, 130.28, 130.03, 129.70, 128.96, 126.60, 125.68, 122.84, 114.32. MS (ESI-TOF) for $C_{16}H_{10}F_3NS$ $[M + H]^+$ calculated 306.0559, found 306.0557.

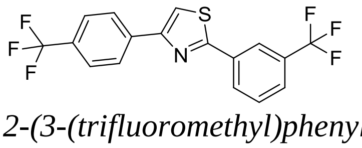

2-(3-(trifluoromethyl)phenyl)-4-(4-(trifluoromethyl)phenyl)thiazole (***4***). White solid (302.1 mg, 80.9 %). m.p. 91.7-95.2 °C. ^{1}H NMR (400 MHz, $CDCl_3$) δ 8.14 (d, J = 8.2 Hz, 2H), 8.07 (dd, J = 7.4, 2.1 Hz, 2H), 7.73 (d, J = 8.3 Hz, 2H), 7.61 (s, 1H), 7.50 (dd, J = 5.0, 2.3 Hz, 3H).

^{13}C NMR (101 MHz, $CDCl_3$) *δ* 168.34, 154.65, 137.65, 133.41, 130.28, 130.03, 129.70, 128.96, 126.60, 125.68, 122.84, 114.32. MS (ESI-TOF) for $C_{17}H_9F_6NS$ $[M + H]^+$ calculated 374.0443, found 374.0447.

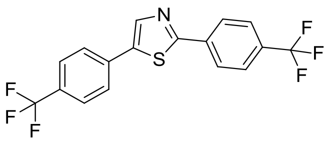

2,5-bis(4-(trifluoromethyl)phenyl)thiazole (***5***). White solid (282 mg, 75.6 %). m.p. 126.3-127.8 °C. ^{1}H NMR (400 MHz, $CDCl_3$) *δ* 8.14 (dd, *J* = 17.3, 8.1 Hz, 96H), 7.74 (t, *J* = 7.3 Hz, 96H), 7.65 (s, 18H), 7.28 (s, 3H). ^{13}C NMR (101 MHz, $CDCl_3$) *δ* 166.40, 155.17, 137.27, 136.42, 131.69, 130.32, 126.77, 125.94, 125.76, 122.75, 115.24. MS (ESI-TOF) for $C_{17}H_9F_6NS$ $[M + H]^+$ calculated 374.0443, found 374.0446.

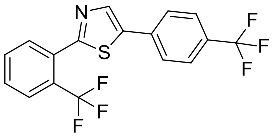

2-(2-(trifluoromethyl)phenyl)-5-(4-(trifluoromethyl)phenyl)thiazole (***6***). White solid (303.1 mg, 81.2 %). m.p. 123.4-126.2 °C. ^{1}H NMR (400 MHz, CDCl3) *δ* 8.11 (d, *J* = 8.2 Hz, 2H), 7.89 (d, *J* = 7.5 Hz, 1H), 7.82 – 7.56 (m, 6H). ^{13}C NMR (101 MHz, CDCl3) *δ* 164.64, 154.31, 137.39, 132.40, 132.14, 131.73, 130.17, 129.84, 129.05, 128.74, 127.00, 126.64, 125.71, 125.49, 124.98, 122.79, 122.26, 116.25. MS (ESI-TOF) for $C_{17}H_9F_6NS$ $[M + H]^+$ calculated 374.0443, found 374.0445.

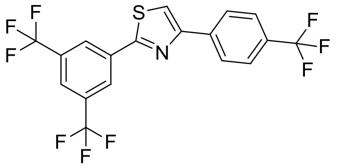

2-(3,5-bis(trifluoromethyl)phenyl)-4-(4-(trifluoromethyl)phenyl)thiazole (***7***). White solid (319.1 mg, 72.3%). m.p. 125.7-126.4 °C. ^{1}H NMR (400 MHz, CDCl3) *δ* 8.49 (s, 2H), 8.15 (d, *J* = 8.1 Hz, 2H), 7.98 (s, 1H), 7.84 – 7.66 (m, 3H). ^{13}C NMR (101 MHz, CDCl3) *δ* 164.58, 155.51, 136.84, 135.20, 132.72, 130.59, 130.27, 127.03, 126.49, 125.78, 125.38, 124.32, 123.38, 122.68, 121.61, 118.89, 115.69. MS (ESI-TOF) for $C_{18}H_8F_9NS$ $[M + H]^+$ calculated 442.0207, found 442.0210.

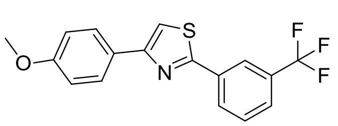

4-(4-methoxyphenyl)-2-(3-(trifluoromethyl)phenyl)thiazole (***8***). White solid (320.58 mg, 95.6 %). m.p. 128.7-129.9 °C. ^{1}H NMR (400 MHz, $CDCl_3$) *δ* 8.12 (d, *J* = 7.7 Hz, 2H), 8.05 – 7.91 (m, 2H), 7.71 (d, *J* = 8.0 Hz, 2H), 7.52 (s, 1H), 7.06 – 6.88 (m, 2H), 3.90 (d, 3H). ^{13}C NMR (101 MHz, $CDCl_3$) *δ* 168.20, 161.33, 154.38, 137.76, 128.09, 126.52, 125.60, 114.27, 113.45, 55.37. MS (ESI-TOF) for $C_{17}H_{12}F_3NOS$ $[M + H]^+$ calculated 336.0664, found 336.0667.

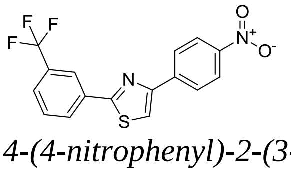

4-(4-nitrophenyl)-2-(3-(trifluoromethyl)phenyl)thiazole (***9***). Yellow solid (349.6 mg, 99.8 %).

m.p. 120.8-121.5 °C. ^{1}H NMR (400 MHz, DMSO) δ 8.51 (s, 1H), 8.26 (dd, J = 33.7, 6.1 Hz, 6H), 7.80 (d, J = 6.3 Hz, 2H). ^{13}C NMR (101 MHz, DMSO) δ 165.01, 154.53, 148.36, 137.46, 127.50, 127.04, 126.03, 124.75, 123.24, 119.59. MS (ESI-TOF) for $C_{16}H_{9}F_{3}N_{2}O_{2}S$ [M + H]$^{+}$ calculated 351.0410, found 351.0415.

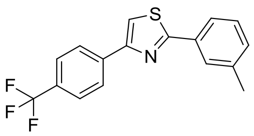

*2-(m-tolyl)-4-(4-(trifluoromethyl)phenyl)thiazole (**10**).* White solid (299.8 mg, 93.9 %). m.p. 94.8-95.5 °C. ^{1}H NMR (400 MHz, $CDCl_3$) δ 8.13 (d, J = 8.0 Hz, 2H), 7.90 (s, 1H), 7.85 (d, J = 7.7 Hz, 1H), 7.72 (d, J = 8.2 Hz, 2H), 7.59 (s, 1H), 7.39 (t, J = 7.6 Hz, 1H), 7.30 (d, J = 7.6 Hz, 1H), 2.48 (s, 3H). ^{13}C NMR (101 MHz, $CDCl_3$) δ 168.59, 154.58, 138.77, 137.69, 133.31, 131.11, 129.99, 128.86, 127.13, 126.56, 125.63, 123.84, 122.85, 114.23, 21.33. MS (ESI-TOF) for $C_{17}H_{14}F_{3}NS$ [M + H]$^{+}$ calculated 332.0872, found 332.0875.

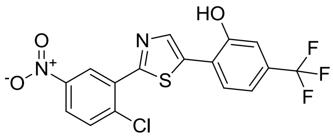

*2-(2-(2-chloro-5-nitrophenyl)thiazol-5-yl)-5-(trifluoromethyl)phenol (**11**).* White solid (560.4 mg, 81.6 %). m.p. 187.4-188.6 °C. ^{1}H NMR (400 MHz, $CDCl_3$) δ 11.54 (s, 1H), 8.93 (d, J = 2.7 Hz, 1H), 8.30 (dd, J = 8.8, 2.7 Hz, 1H), 7.94 (s, 1H), 7.80 (d, J = 8.8 Hz, 2H), 7.35 (d, J = 1.2 Hz, 1H), 7.28 (s, 1H), 7.21 (dd, J = 8.2, 1.2 Hz, 1H). ^{13}C NMR (101 MHz, $CDCl_3$) δ 156.09, 132.32, 126.75, 125.44, 125.10, 116.47. MS (ESI-TOF) for $C_{16}H_{8}ClF_{3}N_{2}O_{3}S$ [M + H]$^{+}$ calculated 400.9969, found 400.9965.

Compound 12 was prepared similar as 2.

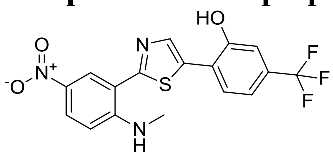

*2-(2-(2-(methylamino)-5-nitrophenyl)thiazol-5-yl)-5-(trifluoromethyl)phenol (**12**).* Yellow solid (192.3 mg, 45.8 %). m.p. 194.6-198.7 °C. ^{1}H NMR (400 MHz, DMSO) δ 11.08 (s, 1H), 9.50 (d, J = 4.8 Hz, 1H), 8.48 (d, J = 2.2 Hz, 1H), 8.39 (s, 1H), 8.27 – 8.10 (m, 2H), 7.29 (d, J = 7.3 Hz, 2H), 6.96 (d, J = 9.4 Hz, 1H), 3.13 (d, 3H). ^{13}C NMR (101 MHz, DMSO) δ 165.55, 155.57, 151.83, 149.65, 135.73, 130.27, 127.26, 125.34, 123.97, 119.10, 116.21, 113.81, 112.90, 111.35, 30.28. MS (ESI-TOF) for $C_{17}H_{12}F_{3}N_{3}O_{3}S$ [M + H]$^{+}$ calculated 396.0624, found 396.0625.

Compounds 13 to 20 were prepared similar as 1.

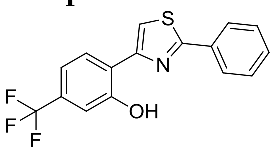

*2-(2-phenylthiazol-4-yl)-5-(trifluoromethyl)phenol (**13**).* White solid (427.6 mg, 90.7 %). m.p. 132.5-134.2 °C. ^{1}H NMR (400 MHz, $CDCl_3$) δ 12.13 (s, 1H), 7.97 – 7.88 (m, 2H), 7.69 (d, J = 8.1 Hz, 1H), 7.60 (s, 1H), 7.50 (s, 3H), 7.32 (s, 1H), 7.14 (d, J = 8.1 Hz, 1H). ^{13}C NMR (101 MHz, $CDCl_3$) δ 168.17, 156.17, 153.04, 131.79, 130.96, 129.17, 126.40, 125.17, 122.51, 122.31, 120.09, 115.78, 115.09, 113.31. MS (ESI-TOF) for $C_{16}H_{10}F_{3}NOS$ [M + H]$^{+}$ calculated 322.0508, found 322.0506.

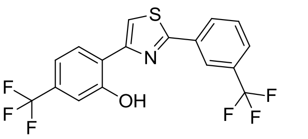

*5-(trifluoromethyl)-2-(2-(3-(trifluoromethyl)phenyl)thiazol-4-yl)phenol (**14**).* White solid (302.5 mg, 74.6 %). m.p. 108.5-109.8 °C. ^{1}H NMR (400 MHz, $CDCl_3$) δ 11.81 (s, 1H), 8.16 (d, J = 7.6 Hz, 2H), 7.76 (dd, J = 13.9, 9.4 Hz, 3H), 7.67 (t, J = 7.8 Hz, 1H), 7.33 (s, 1H), 7.18 (d, J = 8.2 Hz, 1H). ^{13}C NMR (101 MHz, $CDCl_3$) δ 166.43, 156.05, 153.60, 132.60, 129.89, 129.51, 127.42, 126.56, 123.17, 122.15, 119.81, 115.97, 115.16, 114.23. MS (ESI-TOF) for $C_{17}H_9F_6NOS$ $[M + H]^+$ calculated 390.0382, found 390.0378.

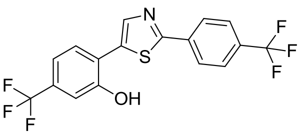

*5-(trifluoromethyl)-2-(2-(4-(trifluoromethyl)phenyl)thiazol-5-yl)phenol (**15**).* White solid (387.6 mg, 70.3 %). m.p. 138.6-139.2 °C. ^{1}H NMR (400 MHz, $CDCl_3$) δ 11.80 (s, 1H), 8.09 (d, J = 8.1 Hz, 2H), 7.88 – 7.69 (m, 4H), 7.34 (s, 1H), 7.19 (d, J = 8.2 Hz, 1H). ^{13}C NMR (101 MHz, $CDCl_3$) δ 166.36, 156.11, 153.77, 134.92, 132.75, 132.34, 132.01, 126.74, 126.45, 125.05, 122.35, 119.80, 116.03, 115.22, 114.50. MS (ESI-TOF) for $C_{17}H_9F_6NOS$ $[M + H]^+$ calculated 390.0382, found 390.0379.

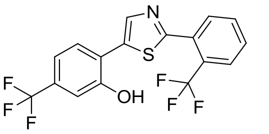

*5-(trifluoromethyl)-2-(2-(2-(trifluoromethyl)phenyl)thiazol-5-yl)phenol (**16**).* White solid (391.3 mg, 71.2 %). m.p. 138.9-140.7 °C. ^{1}H NMR (400 MHz, $CDCl_3$) δ 11.68 (s, 1H), 7.91 (d, J = 7.1 Hz, 1H), 7.83 (s, 1H), 7.78 (d, J = 8.2 Hz, 1H), 7.75 – 7.64 (m, 3H), 7.32 (s, 1H), 7.18 (d, J = 8.2 Hz, 1H). ^{13}C NMR (101 MHz, $CDCl_3$) δ 164.76, 156.17, 153.10, 132.11, 132.02, 130.44, 127.25, 126.54, 119.92, 115.79, 115.41, 115.35. MS (ESI-TOF) for $C_{17}H_9F_6NOS$ $[M + H]^+$ calculated 390.3223, found 390.3005.

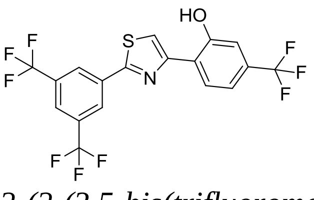

*2-(2-(3,5-bis(trifluoromethyl)phenyl)thiazol-4-yl)-5-(trifluoromethyl)phenol (**17**).* White solid (117.2 mg, 75.6 %). m.p. 157.6-158.2 °C. ^{1}H NMR (400 MHz, $CDCl_3$) δ 11.48 (s, 1H), 8.36 (s, 2H), 8.03 (s, 1H), 7.82 (s, 1H), 7.77 (d, J = 8.2 Hz, 1H), 7.33 (s, 1H), 7.20 (d, J = 8.1 Hz, 1H). ^{13}C NMR (101 MHz, $CDCl_3$) δ 164.62, 155.91, 154.13, 133.82, 133.51, 133.17, 132.49, 132.26, 126.71, 126.24, 124.96, 124.08, 122.25, 121.37, 119.52, 118.66, 116.18, 115.24.MS (ESI-TOF) for $C_{18}H_8F_9NOS$ $[M + H]^+$ calculated 458.0256, found 458.0253.

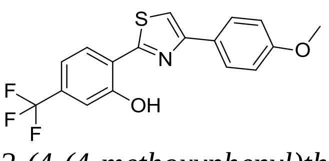

*2-(4-(4-methoxyphenyl)thiazol-2-yl)-5-(trifluoromethyl)phenol (**18**).* White solid (342.7 mg, 86.4 %). m.p. 146.2-147.3 °C. ^{1}H NMR (400 MHz, $CDCl_3$) δ 12.25 (s, 1H), 7.91 (d, J = 8.5 Hz, 2H), 7.74 (d, J = 8.2 Hz, 1H), 7.58 (s, 1H), 7.32 (s, 1H), 7.16 (d, J = 8.1 Hz, 1H), 7.02 (d,

J = 8.6 Hz, 2H), 3.91 (s, 3H). ^{13}C NMR (101 MHz, $CDCl_3$) *δ* 168.10, 161.84, 156.19, 152.75, 131.49, 128.02, 126.37, 124.73, 120.24, 115.76, 115.06, 115.02, 114.50, 112.29, 55.42. MS (ESI-TOF) for $C_{17}H_{12}F_3NO_2S$ $[M + H]^+$ calculated 352.0614, found 352.0610.

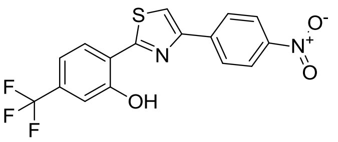

*2-(4-(4-nitrophenyl)thiazol-2-yl)-5-(trifluoromethyl)phenol (***19***).* Yellow solid (186.5 mg, 90.7 %). m.p. 144.9-145.3 °C. ^{1}H NMR (400 MHz, DMSO) *δ* 11.16 (s, 1H), 8.54 (d, *J* = 4.3 Hz, 1H), 8.46 – 8.39 (m, 1H), 8.40 – 8.33 (m, 2H), 8.33 – 8.23 (m, 2H), 7.30 (s, 2H). ^{13}C NMR (101 MHz, DMSO) *δ* 163.54, 157.58, 157.48, 155.61, 151.53, 148.49, 138.56, 132.26, 130.40, 129.76, 127.70, 124.95, 124.00, 122.18, 116.10, 113.10. MS (ESI-TOF) for $C_{16}H_9F_3N_2O_3S$ $[M + H]^+$ calculated 367.0359, found 367.0362.

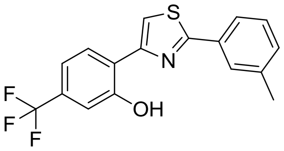

*2-(2-(m-tolyl)thiazol-4-yl)-5-(trifluoromethyl)phenol (***20***).* White solid (277.6 mg, 69.4 %). m.p. 100.7-101.1 °C. ^{1}H NMR (400 MHz, $CDCl_3$) *δ* 12.17 (s, 1H), 7.78 (d, *J* = 6.7 Hz, 3H), 7.65 (d, *J* = 6.1 Hz, 1H), 7.46 – 7.35 (m, 1H), 7.33 (dd, *J* = 13.6, 9.7 Hz, 2H), 7.18 (d, *J* = 7.8 Hz, 1H), 2.48 (d, 3H). ^{13}C NMR (101 MHz, $CDCl_3$) *δ* 168.41, 156.18, 152.96, 139.07, 131.77, 129.06, 126.91, 126.39, 125.18, 123.65, 122.47, 120.15, 115.77, 115.06, 113.16, 21.30. MS (ESI-TOF) for $C_{17}H_{14}F_3NOS$ $[M + H]^+$ calculated 338.0821, found 338.0825.

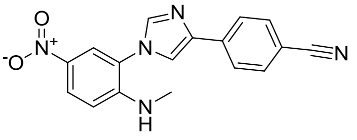

*4-(1-(2-(methylamino)-5-nitrophenyl)-1H-imidazol-4-yl) benzonitrile (***21***).* To a solution of 3-(2-(4-cyanophenyl)-2-oxoethyl)-1-methyl-5-nitro-1H-benzo[d]imidazol-3-ium (100 mg, 0.25 mmol) and glacial acetic acid (100.5 mg, 1.5 mmol) in ammonium acetate (3 mL) was refluxed for 12 h, after which the mixture was added 100ml of distilled water. The resulting yellow precipitate was filtered off to give the crude product and separated in hexane (10 mL). Finally the solid was filtered under reduced pressure to provide the pure product (42.3 mg, 53%). m.p. 171.4-172.6 °C. ^{1}H NMR (400 MHz, DMSO) *δ* 8.24 (dd, *J* = 9.3, 2.6 Hz, 1H), 8.14 (d, *J* = 1.2 Hz, 1H), 8.09 – 8.01 (m, 3H), 7.99 (d, *J* = 1.2 Hz, 1H), 7.91 – 7.77 (m, 2H), 6.87 (d, *J* = 9.4 Hz, 1H), 6.72 (d, *J* = 4.8 Hz, 1H), 2.82 (d, 3H). ^{13}C NMR (101 MHz, DMSO) *δ* 150.63 , 140.20 , 139.61 , 139.12 , 135.47 , 133.04 , 127.15 , 125.33, 124.17, 121.37, 120.20, 119.60, 110.41, 108.98, 30.19. MS (ESI-TOF) for $C_{17}H_{13}N_5O_2$ $[M + H]^+$ calculated 320.3, found 320.1.

Compounds 22 to 26 were prepared similar as 21.

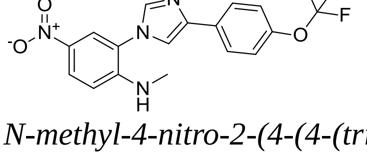

*N-methyl-4-nitro-2-(4-(4-(trifluoromethoxy)phenyl)-1H-imidazol-1-yl)aniline (***22***).* Yellow solid (309.45 mg, 63.7 %). m.p. 194.4-195.1 °C. ^{1}H NMR (400 MHz, DMSO) *δ* 8.23 (dd, *J* = 9.2, 2.5 Hz, 1H), 8.02 (d, *J* = 2.7 Hz, 1H), 8.01 – 7.92 (m, 4H), 7.40 (dd, *J* = 8.9, 0.9 Hz, 2H), 6.87 (d, *J* = 9.4 Hz, 1H), 6.71 (q, *J* = 4.5 Hz, 1H), 2.83 (s, 3H). ^{13}C NMR (101 MHz, DMSO)

δ 150.69, 147.38, 140.61, 139.15, 135.55, 134.04, 127.08, 126.54, 124.11, 121.89, 121.67, 121.63, 119.35, 118.28, 110.45, 30.27. MS (ESI-TOF) for $C_{17}H_{13}F_3N_4O_3$ $[M + H]^+$ calculated 379.1013, found 379.1013.

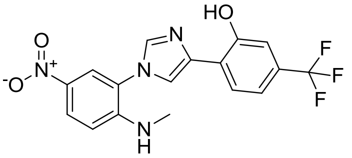

*2-(1-(2-(methylamino)-5-nitrophenyl)-1H-imidazol-4-yl)-5-(trifluoromethyl) phenol (**23**).* Provide the pure product as yellow solid (35 mg, 50%). m.p. 198.3-199.2 °C. ^{1}H NMR (400 MHz, DMSO) δ 11.83 (s, 1H), 8.25 (dd, J = 9.3, 2.6 Hz, 1H), 8.13 (d, J = 1.0 Hz, 1H), 8.10 – 7.97 (m, 3H), 7.21 (d, J = 8.0 Hz, 2H), 6.88 (d, J = 9.4 Hz, 1H), 6.73 (d, J = 4.8 Hz, 1H), 2.82 (d, 3H). ^{13}C NMR (101 MHz, DMSO) δ 155.24), 150.74, 138.46, 137.90, 135.48, 128.30, 127.25, 126.96, 125.95, 124.43, 122.83, 121.23, 120.09, 115.89, 113.10, 110.44, 30.17. MS (ESI-TOF) for $C_{17}H_{13}F_3N_4O_3$ $[M + H]^-$ calculated 379.1013, found 379.1012.

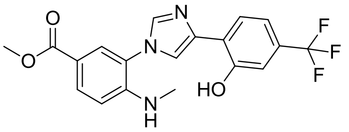

*methyl 3-(4-(2-hydroxy-4-(trifluoromethyl)phenyl)-1H-imidazol-1-yl)-4-(methylamino)benzoate (**25**).* Gray solid (64mg, 34%). m.p. 182.2-183.5 °C. ^{1}H NMR (400 MHz, DMSO) δ 11.99 (s, 1H), 8.14 – 7.98 (m, 3H), 7.94 (dd, J = 8.7, 1.5 Hz, 1H), 7.68 (d, J = 1.8 Hz, 1H), 7.21 (d, J = 6.9 Hz, 2H), 6.84 (d, J = 8.8 Hz, 1H), 6.12 (d, J = 4.8 Hz, 1H), 3.80 (s, 3H), 2.77 (d, 3H). ^{13}C NMR (101 MHz, DMSO) δ 166.06, 155.36, 148.95, 137.76, 132.25, 129.03, 126.87, 122.68, 121.74, 119.95, 116.16, 113.17, 110.67, 51.96, 29.95. MS (ESI-TOF) for $C_{19}H_{16}F_3N_3O_3$ $[M + H]^+$ calculated 392.1217, found 392.1215.

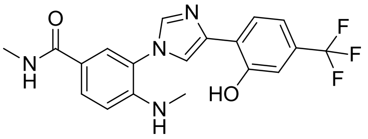

*3-(4-(2-hydroxy-4-(trifluoromethyl)phenyl)-1H-imidazol-1-yl)-N-methyl-4-(methylamino)benzamide (**26**).* Gray solid (64mg, 34%). m.p. 194.9-195.6 °C. ^{1}H NMR (400 MHz, DMSO) δ 12.00 (s, 1H), 8.19 (d, J = 4.5 Hz, 1H), 8.13 – 7.97 (m, 3H), 7.88 (dd, J = 8.6, 1.9 Hz, 1H), 7.70 (d, J = 2.0 Hz, 1H), 7.21 (d, J = 6.8 Hz, 2H), 6.80 (d, J = 8.7 Hz, 1H), 5.78 (d, 1H), 2.75 (t, 6H). 13C NMR (101 MHz, DMSO) δ 166.00, 155.33, 147.17, 138.34, 137.79, 130.01, 128.26, 126.86, 125.97, 123.27, 122.73, 121.52, 120.09, 115.87, 113.13, 110.50, 30.07, 26.52. MS (ESI-TOF) for $C_{19}H_{17}F_3N_4O_2$ $[M + H]^+$ calculated 391.1376, found 391.1375.

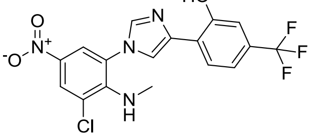

*2-(1-(3-chloro-2-(methylamino)-5-nitrophenyl)-1H-imidazol-4-yl)-5-(trifluoromethyl)phenol (**27**).* To a mixture of 2-(1-(2-(methylamino)-5-nitrophenyl)-1H-imidazol-4-yl)-5-(trifluoromethyl)phenol (30 mg, 0.08 mmol) in sulfurous dichloride (4 mL) was reacted in 0 °C. Then the reaction was monitored by TLC. After reaction the solvent was evaporated under reduced pressure and the solid residue was purified by flash column chromatography on

silica gel (PE-EA=2:1) as eluent to provide 2-(1-(3-chloro-2-(methylamino)-5-nitrophenyl)-1H-imidazol-4-yl)-5-(trifluoromethyl)phenol as yellow solid (28.39 mg, 86%). m.p. 130.8-131.9 °C. ^{1}H NMR (400 MHz, DMSO) δ 11.53 (s, 1H), 8.31 (d, *J* = 2.7 Hz, 1H), 8.21 (d, *J* = 1.1 Hz, 1H), 8.16 – 8.06 (m, 3H), 7.21 (d, *J* = 6.4 Hz, 2H), 6.87 (d, *J* = 5.3 Hz, 1H), 2.40 (d, 3H). ^{13}C NMR (101 MHz, DMSO) δ 155.06, 147.76, 139.87, 137.31, 135.04, 128.39, 128.08, 127.21, 126.37, 125.78, 122.93, 120.51, 120.11, 115.95, 112.88, 30.06. MS (ESI-TOF) for $C_{17}H_{12}ClF_3N_4O_3$ [M + H]$^+$ calculated 413.0623, found 413.0625.

Compound 28 was prepared similar as 27.

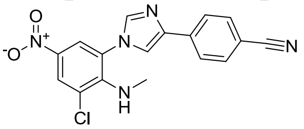

*4-(1-(3-chloro-2-(methylamino)-5-nitrophenyl)-1H-imidazol-4-yl) benzonitrile (**28**).* Yellow solid (34.2 mg, 62%). m.p. 210.8-212.6 °C. ^{1}H NMR (400 MHz, DMSO) δ 8.31 (d, *J* = 2.5 Hz, 1H), 8.25 (s, 1H), 8.11 (s, 1H), 8.08 (d, *J* = 2.4 Hz, 1H), 8.02 (d, *J* = 8.2 Hz, 2H), 7.85 (d, *J* = 8.2 Hz, 2H), 6.89 (s, 1H), 2.41 (s, 3H). ^{13}C NMR (101 MHz, DMSO) δ 147.70, 141.57, 139.64, 138.83, 135.03, 133.09, 126.26, 125.77, 125.39, 122.46, 120.56, 120.08, 119.55, 109.23, 30.20. MS (ESI-TOF) for $C_{17}H_{12}ClN_5O_2$ [M + H]$^+$ calculated 354.0752, found 354.0753.

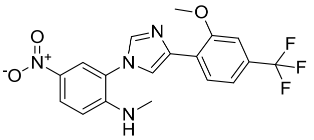

*2-(4-(2-methoxy-4-(trifluoromethyl) phenyl)-1H-imidazol-1-yl)-N-methyl-4-nitroaniline (**29**).* To a mixture of compound **23** (30 mg, 0.082 mmol) with anhydrous potassium carbonate (101.8 mg, 0.738 mmol) in acetone (3 mL). After 15 minutes the solution was added iodomethane (34.9 mg, 0.246 mmol) dropwise. The mixture was reacted at 50 °C for 24 hours and was monitored by TLC. Then the crude mixture was stopped under concentrated pressure and diluted with water then extracted sequentially with ethyl acetate (3 × 50 mL). The organic layer was collected and dried over Na_2SO_4. The organic solution was again concentrated to dryness. The crude mixture was purified by column chromatography to afford the product as yellow solid (28.6 mg, 92%). m.p. 201.5-202.2 °C. ^{1}H NMR (400 MHz, DMSO) δ 8.39 (d, *J* = 8.0 Hz, 1H), 8.23 (dd, *J* = 9.3, 2.7 Hz, 1H), 8.01 (d, *J* = 2.7 Hz, 1H), 7.97 (d, *J* = 1.2 Hz, 1H), 7.88 (d, *J* = 1.1 Hz, 1H), 7.40 (d, *J* = 8.2 Hz, 1H), 7.35 (s, 1H), 6.87 (d, *J* = 9.4 Hz, 1H), 6.67 (q, *J* = 4.5 Hz, 1H), 3.99 (s, 3H), 2.82 (d, 3H). ^{13}C NMR (101 MHz, DMSO) δ 156.00, 150.93, 138.41, 136.21, 135.45, 128.22, 127.96, 127.65, 127.33, 127.11, 126.79, 126.12, 124.43, 123.42, 122.35, 121.53, 117.59, 110.33, 108.13, 56.16, 30.18. MS (ESI-TOF) for $C_{18}H_{15}F_3N_4O_3$ [M + H]$^+$ calculated 393.1169, found 393.1166.

Compounds 30 to 35 were prepared similar as 29.

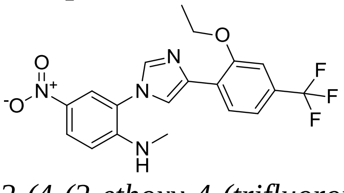

*2-(4-(2-ethoxy-4-(trifluoromethyl)phenyl)-1H-imidazol-1-yl)-N-methyl-4-nitroaniline (**30**).* Yellow solid (95 mg, 82 %). m.p. 208.8-209.8 °C. ^{1}H NMR (400 MHz, DMSO) δ 8.39 (d, *J* = 8.0 Hz, 1H), 8.23 (dd, *J* = 9.3, 2.6 Hz, 1H), 8.00 (dd, *J* = 9.9, 1.8 Hz, 2H), 7.81 (d, *J* = 0.8 Hz, 1H), 7.39 (d, *J* = 8.2 Hz, 1H), 7.33 (s, 1H), 6.88 (d, *J* = 9.4 Hz, 1H), 6.68 (d, *J* = 4.8 Hz, 1H), 4.27 (q, 2H), 2.83 (d, 3H), 1.42 (t, *J* = 6.9 Hz, 3H). ^{13}C NMR (101 MHz, DMSO) δ 155.19,

150.77, 138.43, 136.35, 135.54, 127.55, 127.05, 126.78, 124.19, 121.83, 121.54, 117.53, 110.44, 108.86, 64.59, 30.19, 14.88. MS (ESI-TOF) for $C_{19}H_{17}F_3N_4O_3$ $[M + H]^+$ calculated 407.1326, found 407.1325.

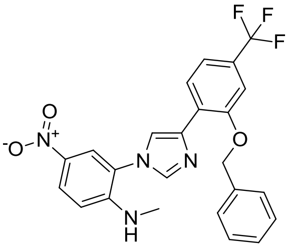

*2-(4-(2-(benzyloxy)-4-(trifluoromethyl)phenyl)-1H-imidazol-1-yl)-N-methyl-4-nitroaniline (**31**).* Yellow solid (159 mg, 92 %). m.p. 217.3-218.4 °C. ^{1}H NMR (400 MHz, DMSO) δ 8.40 (d, J = 8.0 Hz, 1H), 8.21 (dd, J = 9.3, 2.6 Hz, 1H), 7.99 (dd, J = 16.5, 2.0 Hz, 2H), 7.77 (d, J = 1.1 Hz, 1H), 7.54 (d, J = 6.9 Hz, 2H), 7.46 (s, 1H), 7.41 (d, J = 8.1 Hz, 1H), 7.39 – 7.24 (m, 3H), 6.85 (d, J = 9.4 Hz, 1H), 6.72 (d, J = 4.8 Hz, 1H), 5.39 (s, 2H), 2.82 (d, 3H). ^{13}C NMR (101 MHz, DMSO) δ 154.90, 150.48, 138.34, 136.92, 136.26, 135.50, 128.85, 128.50, 128.30, 127.53, 127.12, 126.97, 123.84, 122.05, 121.46, 117.91, 110.38, 109.72, 70.53, 30.24. MS (ESI-TOF) for $C_{24}H_{19}F_3N_4O_3$ $[M + H]^+$ calculated 469.1482, found 469.1485.

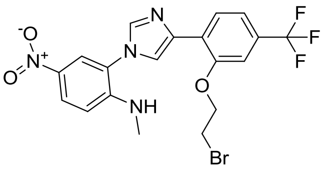

*2-(4-(2-(2-bromoethoxy)-4-(trifluoromethyl)phenyl)-1H-imidazol-1-yl)-N-methyl-4-nitroaniline (**32**).* Yellow solid (109 mg, 90 %). m.p. 209.3-210.5 °C. ^{1}H NMR (400 MHz, DMSO) δ 8.40 (d, J = 8.0 Hz, 1H), 8.22 (d, J = 9.2 Hz, 1H), 8.07 (s, 1H), 8.03 (s, 2H), 7.49 – 7.31 (m, 2H), 6.88 (d, J = 9.2 Hz, 1H), 6.74 (d, J = 3.5 Hz, 1H), 4.58 (s, 2H), 3.98 (s, 2H), 2.83 (d, 3H). ^{13}C NMR (101 MHz, DMSO) δ 154.55, 150.50, 138.34, 135.99, 135.50, 128.00, 127.59, 126.95, 126.02, 123.88, 123.32, 122.51, 121.56, 118.13, 110.42, 109.20, 68.99, 32.13, 30.22. MS (ESI-TOF) for $C_{19}H_{16}BrF_3N_4O_3$ $[M + H]^+$ calculated 485.0431, found 485.0435.

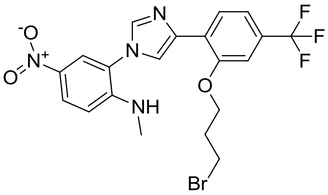

*2-(4-(2-(3-bromopropoxy)-4-(trifluoromethyl)phenyl)-1H-imidazol-1-yl)-N-methyl-4-nitroaniline (**33**).* Yellow solid (153 mg, 90 %). m.p. 213.3-214.8 °C. ^{1}H NMR (400 MHz, DMSO) δ 8.39 (d, J = 7.8 Hz, 1H), 8.23 (d, J = 9.1 Hz, 1H), 8.02 (s, 2H), 7.82 (s, 1H), 7.41 (d, J = 13.2 Hz, 2H), 6.87 (d, J = 9.2 Hz, 1H), 6.73 (s, 1H), 4.34 (s, 2H), 3.70 (d, 2H), 2.83 (d, 3H), 2.45 – 2.21 (m, 2H). ^{13}C NMR (101 MHz, DMSO) δ 154.99, 150.76, 138.48, 136.19, 135.57, 127.79, 127.75, 124.13, 121.95, 121.61, 117.92, 117.88, 110.47, 109.05, 66.90, 32.09, 31.83, 30.28. MS (ESI-TOF) for $C_{20}H_{18}BrF_3N_4O_3$ $[M + H]^+$ calculated 499.0587, found 499.0585.

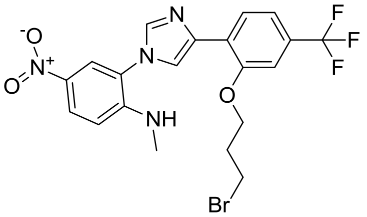

*2-(4-(2-(3-bromopropoxy)-4-(trifluoromethyl)phenyl)-1H-imidazol-1-yl)-N-methyl-4-nitroaniline (**34**).* Yellow solid (178 mg, 92 %). m.p. 188.1-189.9 °C. ^{1}H NMR (400 MHz, DMSO) δ 8.38 (d, J = 8.0 Hz, 1H), 8.23 (dd, J = 9.3, 2.5 Hz, 1H), 8.06 – 7.92 (m, 3H), 7.46 – 7.31 (m, 2H), 6.86 (d, J = 9.4 Hz, 1H), 6.67 (d, J = 4.7 Hz, 1H), 4.39 – 4.25 (m, 2H), 3.92 – 3.81 (m, 2H), 3.73 (t, 2H), 3.42 (t, 2H), 2.82 (d, 3H). ^{13}C NMR (101 MHz, DMSO) δ 155.16, 150.95, 138.46, 136.31, 135.53, 127.47, 127.16, 126.14, 124.36, 123.44, 122.53, 121.62, 117.85, 110.44, 109.14, 70.64, 68.94, 67.99, 32.11, 30.26. MS (ESI-TOF) for $C_{21}H_{20}BrF_3N_4O_4$ $[M + H]^+$ calculated 499.0587, found 499.0585.

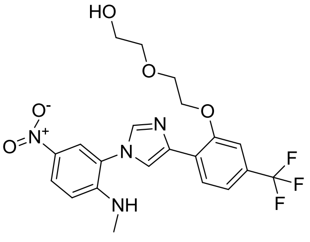

*2-(2-(2-(1-(2-(methylamino)-5-nitrophenyl)-1H-imidazol-4-yl)-5 (trifluoromethyl)phenoxy)ethoxy)ethanol (**35**).* Yellow solid (141 mg, 76 %). m.p. 172.5-173.6 °C. ^{1}H NMR (400 MHz, DMSO) δ 8.37 (d, J = 7.9 Hz, 1H), 8.23 (dd, J = 9.2, 2.2 Hz, 1H), 7.99 (t, J = 4.3 Hz, 3H), 7.40 (d, J = 10.8 Hz, 2H), 6.87 (d, J = 9.3 Hz, 1H), 6.68 (d, J = 4.7 Hz, 1H), 4.46 (t, 1H), 4.33 (d, 2H), 3.84 (d, 2H), 3.42 (t, 2H), 3.32 (s, 2H), 2.83 (d, 3H). ^{13}C NMR (101 MHz, DMSO) δ 155.18, 150.69, 138.32, 136.30, 135.50, 127.35, 124.03, 122.49, 121.57, 117.79, 110.38, 72.66, 69.24, 68.08, 60.49, 30.19. MS (ESI-TOF) for $C_{21}H_{21}F_3N_4O_5$ $[M + H]^+$ calculated 467.1537, found 467.1535.

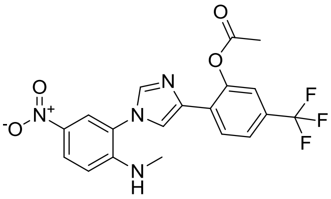

*2-(1-(2-(methylamino)-5-nitrophenyl)-1H-imidazol-4-yl)-5-(trifluoromethyl)phenyl acetate (**36**).* To a mixture of compound **23** (10 mg, 0.026 mmol) and triethylamine (200 uL) in dichloromethane (2 ml) in 0 °C was added by acetyl chloride (200 uL). Then the reaction was reacted for 1 hour and monitored by TLC board. Once stopped, the solvent was evaporated under reduced pressure. The crude mixture was purified by column chromatography (PE-EA=2:1) to afford the product(293.3 mg, 85.1 %). m.p. 134.7-136.1 °C. ^{1}H NMR (400 MHz, DMSO) δ 8.34 (d, J = 8.2 Hz, 1H), 8.23 (dd, J = 9.3, 2.6 Hz, 1H), 8.03 (d, J = 2.7 Hz, 2H), 7.83 (s, 1H), 7.75 – 7.62 (m, 2H), 6.88 (d, J = 9.3 Hz, 1H), 6.73 (d, J = 4.7 Hz, 1H), 2.82 (d, 3H), 2.38 (s, 3H). ^{13}C NMR (101 MHz, DMSO) δ 169.42, 150.75, 146.94, 139.09, 135.54, 130.99, 128.81, 127.07, 124.32, 122.96, 121.41, 110.43, 30.17, 21.71. MS (ESI-TOF) for $C_{19}H_{15}F_3N_4O_4$ $[M + H]^+$ calculated 421.1118, found 421.1115.

Compounds 37 to 40 were prepared similar as 36.

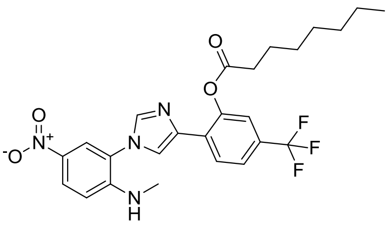

*2-(1-(2-(methylamino)-5-nitrophenyl)-1H-imidazol-4-yl)-5-(trifluoromethyl)phenyl octanoate (**37**).* Yellow solid (105 mg, 91 %). m.p. 150.6-152.2 °C. ^{1}H NMR (400 MHz, DMSO) δ 8.34 (d, J = 8.2 Hz, 1H), 8.23 (dd, J = 9.3, 2.4 Hz, 1H), 8.05 – 7.95 (m, 2H), 7.77 (s, 1H), 7.70 (d, J = 8.3 Hz, 1H), 7.62 (s, 1H), 6.87 (d, J = 9.3 Hz, 1H), 6.74 (d, J = 4.7 Hz, 1H), 2.82 (d, 3H), 2.71 (t, 2H), 1.70 – 1.55 (m, 2H), 1.26 (ddd, 8H), 0.82 (t, 3H). ^{13}C NMR (101 MHz, DMSO) δ 171.95, 150.68, 146.97, 139.05, 135.86, 135.52, 131.04, 128.81, 127.85, 127.53, 127.06, 125.60, 124.17, 122.99, 121.38, 110.44, 34.06, 31.41, 30.14, 28.73, 24.40, 22.40, 14.22. MS (ESI-TOF) for $C_{25}H_{27}F_{3}N_{4}O_{4}$ $[M + H]^{+}$ calculated 505.2057, found 505.2059.

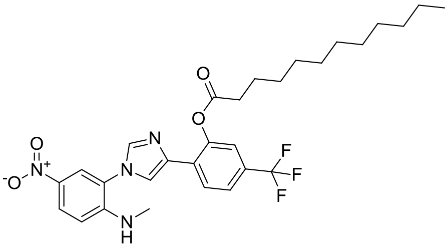

*2-(1-(2-(methylamino)-5-nitrophenyl)-1H-imidazol-4-yl)-5-(trifluoromethyl)phenyl dodecanoate (**38**).* Yellow solid (154 mg, 90 %). m.p. 158.3-159.6 °C. ^{1}H NMR (400 MHz, DMSO) δ 8.34 (d, J = 8.2 Hz, 1H), 8.21 (dd, J = 9.2, 2.1 Hz, 1H), 8.05 – 7.94 (m, 2H), 7.76 (s, 1H), 7.69 (d, J = 8.3 Hz, 1H), 7.61 (s, 1H), 6.87 (d, J = 9.3 Hz, 1H), 6.75 (d, J = 4.6 Hz, 1H), 2.81 (d, 3H), 2.70 (t, 2H), 1.69 – 1.54 (m, 2H), 1.27 – 1.12 (m, 17H), 0.84 (t, 4H). ^{13}C NMR (101 MHz, DMSO) δ 171.91, 150.66, 146.96, 139.02, 135.86, 135.51, 131.02, 128.81, 127.85, 127.53, 127.03, 125.59, 124.13, 123.01, 121.35, 121.31, 121.09, 110.42, 34.05, 31.69, 30.12, 29.37, 29.17, 29.11, 29.04, 28.78, 24.38, 22.49, 14.28. MS (ESI-TOF) for $C_{29}H_{35}F_{3}N_{4}O_{4}$ $[M + H]^{+}$ calculated 561.2683, found 561.2683.

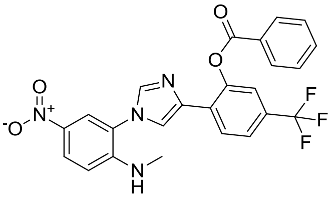

*2-(1-(2-(methylamino)-5-nitrophenyl)-1H-imidazol-4-yl)-5-(trifluoromethyl)phenyl benzoate (**39**).* Yellow solid (186 mg, 90 %). m.p. 203.3-204.3 °C. ^{1}H NMR (400 MHz, DMSO) δ 8.37 (d, J = 8.3 Hz, 1H), 8.22 (t, J = 7.9 Hz, 2H), 8.14 (d, J = 9.2 Hz, 1H), 8.00 (d, J = 9.3 Hz, 1H), 7.89 – 7.80 (m, 2H), 7.75 (dd, J = 18.0, 8.6 Hz, 2H), 7.65 (d, J = 9.1 Hz, 1H), 7.60 (t, J = 7.8 Hz, 2H), 6.77 (d, J = 9.3 Hz, 1H), 6.62 (s, 1H), 2.65 (d, 3H). ^{13}C NMR (101 MHz, DMSO) δ 164.63, 150.23, 147.06, 139.06, 135.91, 135.46, 134.55, 131.23, 130.37, 129.39, 129.26, 128.99, 126.95, 123.73, 121.15, 110.40, 30.04. MS (ESI-TOF) for $C_{24}H_{17}F_{3}N_{4}O_{4}$ $[M + H]^{+}$ calculated 483.1275, found 483.1271.

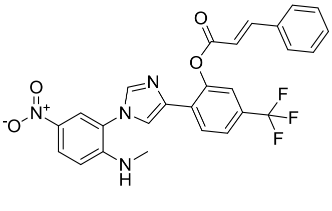

*2-(1-(2-(methylamino)-5-nitrophenyl)-1H-imidazol-4-yl)-5-(trifluoromethyl)phenyl cinnamate (**40**).* Yellow solid (119 mg, 85 %). m.p. 209.3-210.2 °C. ^{1}H NMR (400 MHz, DMSO) δ 8.40 (d, J = 8.1 Hz, 1H), 8.16 (d, J = 9.2 Hz, 1H), 8.03 (s, 1H), 7.98 (s, 1H), 7.93 (d, J = 16.0 Hz, 1H), 7.85 (s, 1H), 7.82 – 7.68 (m, 4H), 7.44 (d, J = 6.8 Hz, 3H), 7.07 (d, J = 16.0 Hz, 1H), 6.77 (d, J = 9.3 Hz, 1H), 6.67 (d, J = 4.6 Hz, 1H), 2.67 (d, 3H). ^{13}C NMR (101 MHz, DMSO) δ 164.92, 150.52, 147.46, 146.88, 139.11, 135.80, 135.50, 134.27, 131.35, 131.15, 129.31, 129.09, 128.82, 127.90, 127.58, 127.01, 124.04, 123.18, 121.52, 121.30, 117.65, 110.38, 30.07. MS (ESI-TOF) for $C_{26}H_{19}F_3N_4O_4$ [M + H]$^+$ calculated 509.1431, found 509.1435.

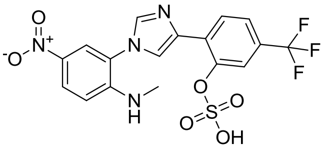

*2-(1-(2-(methylamino)-5-nitrophenyl)-1H-imidazol-4-yl)-5-(trifluoromethyl)phenyl hydrogen sulfate (**41**).* To a mixture of compound **23** (18.9 mg, 0.05 mmol), triethylamine (400 uL)and Pyridine - Sulfur Trioxide Complex (40 mg, 0.25 mmol) in anhydrous THF (5 mL). Then the mixture was reacted in reflux temperature for 4 hours and monitored by TLC. Then the crude mixture was stopped under concentrated pressure and extracted sequentially with ethyl acetate (3 × 50 mL). The organic layer was collected and dried over Na_2SO_4. The organic solution was again concentrated to dryness. The crude mixture was purified by column chromatography (EA-MeOH=19:1) to afford the product as yellow solid (21.6 mg, 94.6 %). m.p. 173.3-175.6 °C. ^{1}H NMR (400 MHz, DMSO) δ 8.36 (d, J = 8.2 Hz, 1H), 8.24 (dd, J = 9.3, 2.6 Hz, 1H), 8.08 – 7.95 (m, 2H), 7.89 (d, J = 20.3 Hz, 2H), 7.49 (d, J = 7.8 Hz, 1H), 6.88 (d, J = 9.4 Hz, 1H), 6.65 (d, J = 4.8 Hz, 1H), 2.83 (d, 3H). ^{13}C NMR (101 MHz, DMSO) δ 150.73, 150.19, 138.37, 136.28, 135.51, 129.33, 127.44, 127.18, 124.09, 122.23, 121.41, 119.86, 117.08, 110.50, 30.20. MS (ESI-TOF) for $C_{17}H_{13}F_3N_4O_6S$ [M + H]$^-$calculated 459.0581, found 459.0579.

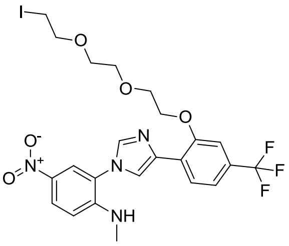

*2-(4-(2-(2-(2-iodoethoxy) ethoxy) ethoxy)-4-(trifluoromethyl) phenyl)-1H-imidazol-1-yl)-N-methyl-4-nitroaniline (**42**).* To a stired mixture of compound **23** (36 mg, 0.1 mmol) with potassium carbonate (124 mg, 0.9 mmol) in acetone (2 mL). After 15 minutes the solution was added 1,2-bis(2-iodoethoxy) ethane (27.3 mg, 0.15 mmol) inside. The mixture was reacted at 45 °C for 24 hours and was monitored by TLC. Then the crude mixture was stopped under concentrated pressure and extracted sequentially with ethyl acetate (3 × 50 mL). The organic layer was collected and dried over Na_2SO_4. The organic solution was again concentrated to dryness. The crude mixture was purified by column chromatography to afford the product as yellow solid (39 mg, 80%). m.p. 166.7-168.2 °C. ^{1}H NMR (400 MHz, $CDCl_3$) δ 8.33 (d, J = 7.0 Hz, 2H), 8.16 (d, J = 2.4 Hz, 1H), 7.97 (s, 1H), 7.82 (s, 1H), 7.34 (d, J = 7.9 Hz, 1H), 7.17 (s, 1H), 6.81 (d, J = 9.3 Hz, 1H), 5.19 (s, 1H), 4.39 – 4.24 (m, 2H), 4.03 – 3.88 (m, 2H), 3.72 – 3.62 (m, 2H), 3.58 (t, 2H), 3.55 – 3.47 (m, 2H), 3.13 (t, 2H), 3.01 (d, 3H). ^{13}C NMR (101 MHz, $CDCl_3$) δ 154.82, 149.79, 136.90, 136.53, 127.34, 127.01, 125.43, 123.71, 121.49, 109.50, 71.72, 70.16, 69.48, 67.31, 30.02, 2.66. MS (ESI-TOF) for $C_{23}H_{24}F_3IN_4O_5$ [M + H]$^+$ calculated 621.0816, found 621.0820.

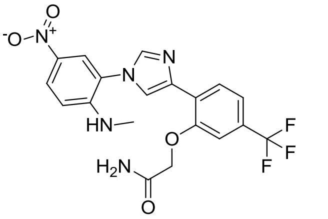

*2-(2-(1-(2-(methylamino)-5-nitrophenyl)-1H-imidazol-4-yl)-5-(trifluoromethyl) phenoxy) acetamide (**43**).* To a solution of compound **23** (30 mg, 0.082 mmol) and anhydrous potassium carbonate (25.5 mg, 0.184 mmol) and potassium iodide (1.369 mg, 0.0082 mmol) in 2-butanone (2.5 mL) were refluxed for 30 minutes. The solution was added by 2-chloroacetamide (9.99 mg, 0.102 mmol) and the reaction continued for 12 hours. Once stopped, the solvent was evaporated under reduced pressure. Then the crude mixture was diluted with water and extracted sequentially with ethyl acetate (3 × 50 mL). The organic layer was collected and dried over Na_2SO_4. The organic solution was again concentrated to dryness. The crude mixture was purified by column chromatography to afford the product as yellow solid (31.3 mg, 90.3%). m.p. 226.7-228.9 °C. ^{1}H NMR (400 MHz, DMSO) δ 8.37 (d, J = 8.0 Hz, 1H), 8.24 (dd, J = 9.3, 2.6 Hz, 1H), 8.19 (s, 1H), 8.05 – 7.96 (m, 2H), 7.59 (s, 1H), 7.43 (d, J = 8.2 Hz, 1H), 7.35 (s, 1H), 7.24 (s, 1H), 6.88 (d, J = 9.4 Hz, 1H), 6.68 (d, J = 4.7 Hz, 1H), 4.74 (s, 2H), 2.83 (d, 3H). ^{13}C NMR (101 MHz, DMSO) δ 169.81, 154.54, 150.78, 138.40, 136.24, 135.52, 127.69, 127.13, 124.20, 123.00, 121.61, 110.44, 99.98, 67.58, 30.26. MS (ESI-TOF) for $C_{19}H_{16}F_3N_5O_4$ $[M + H]^+$ calculated 436.1227, found 436.1227.

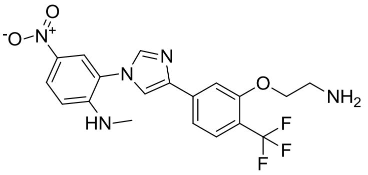

*2-(4-(3-(2-aminoethoxy)-4-(trifluoromethyl)phenyl)-1H-imidazol-1-yl)-N-methyl-4-nitroaniline (**44**).* To a mixture of compound **32** (35 mg, 0.082 mmol), potassium iodide (68 mg, 0.41 mmol) was dissolved with tetrahydrofuran (4 mL), and then ammonium hydroxide aqueous solution (30%, 4 mL) was added. The reaction was heated to 70 °C and stirred in a sealed tube for 24 h. After cooling down, the reaction was monitored by TLC board. Once stopped, the aqueous layer was extracted with ethyl acetate (3×15 mL). The organic layers were combined, dried over Na_2SO_4 and purified by column chromatography (EA-MeOH=15:1) to afford the yellow product (20.7 mg, 60.1 %). m.p. 136.9-139.5 °C. ^{1}H NMR (400 MHz, $CDCl_3$) δ 8.23 (dd, J = 9.2, 2.4 Hz, 1H), 8.15 – 7.97 (m, 2H), 7.61 (d, J = 8.0 Hz, 1H), 7.38 (s, 1H), 7.33 (d, J = 8.2 Hz, 1H), 7.25 (s, 1H), 6.72 (d, J = 9.3 Hz, 1H), 5.04 (d, 1H), 4.54 (s, 2H), 3.51 (s, 2H), 2.96 (d, 3H). ^{13}C NMR (101 MHz, DMSO) δ 154.97, 150.74, 138.40, 136.16, 135.52, 127.91, 127.22, 127.01, 126.03, 124.16, 123.33, 122.28, 121.59, 118.05, 110.44, 109.44, 68.90, 30.21, 22.88. MS (ESI-TOF) for $C_{19}H_{18}F_3N_5O_3$ $[M + H]^+$ calculated 422.1435, found 422.1430.

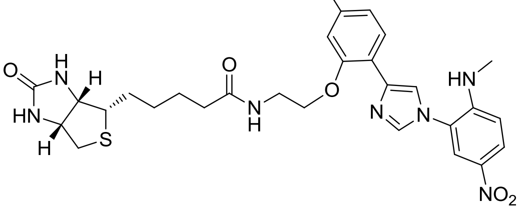

*N-(2-(2-(1-(2-(methylamino)-5-nitrophenyl)-1H-imidazol-4-yl)-5-(trifluoromethyl)phenoxy)ethyl)-5-((3aS,4S,6aR)-2-oxohexahydro-1H-thieno[3,4-d]imidazol-4-yl)pentanamide (**45**).* To a solution of HATU (80.94 mg, 0.213 mmol), DIPEA (2 mL) and

D-Biotin (34.8 mg, 0.14 mmol) in DMF (4 mL). After 30 minutes was added 2-(4-(2-(2-aminoethoxy)-4-(trifluoromethyl) phenyl)-1H-imidazol-1-yl)-N-methyl-4-nitroaniline (30 mg, 0.071 mmol). The mixture was reacted at 45 °C for 8 hours and was monitored by TLC. Then the crude mixture was stopped under concentrated pressure of oil pump and extracted sequentially with ethyl acetate (3 × 50 mL). The organic layer was collected and dried over Na_2SO_4. The organic solution was again concentrated to dryness. The crude mixture was purified by column chromatography to afford the product as yellow solid (42.76 mg, 93%). m.p. 118.7-120.5 °C. ^{1}H NMR (400 MHz, $CDCl_3$) δ 8.34 – 8.19 (m, 2H), 8.12 (d, J = 2.4 Hz, 1H), 7.74 (d, J = 25.4 Hz, 2H), 7.33 (d, J = 8.2 Hz, 1H), 7.14 (dd, J = 13.3, 8.0 Hz, 2H), 6.76 (d, J = 9.3 Hz, 1H), 6.31 (s, 1H), 5.28 (d, J = 4.3 Hz, 1H), 5.12 (s, 1H), 4.35 – 4.27 (m, 1H), 4.24 (t, 2H), 4.13 – 4.04 (m, 1H), 3.75 (d, 2H), 2.98 (d, 4H), 2.78 (dd, 1H), 2.54 (d, 1H), 2.11 (t, 2H), 1.50 (d, 4H), 1.28 (d, 3H). ^{13}C NMR (101 MHz, DMSO) δ 172.71, 163.12, 155.07, 150.90, 138.36, 136.06, 135.44, 127.57, 124.42, 121.62, 117.76, 110.33, 108.93, 79.62, 67.67, 61.40, 59.59, 55.82, 53.74, 42.03, 38.31, 35.56, 31.37, 30.22, 28.55, 25.41, 22.47, 14.36, 12.75. MS (ESI-TOF) for $C_{29}H_{32}F_3N_7O_5S$ $[M + H]^+$ calculated 648.2210, found 648.2206.

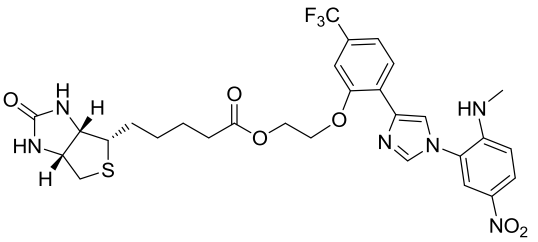

*2-(2-(1-(2-(methylamino)-5-nitrophenyl)-1H-imidazol-4-yl)-5-(trifluoromethyl)phenoxy)ethyl 5-((3aS,4S,6aR)-2-oxohexahydro-1H-thieno[3,4-d]imidazol-4-yl)pentanoate (**46**).* Yellow solid (67 mg, 71 %). m.p. 79.1-80.5 °C. ^{1}H NMR (400 MHz, $CDCl_3$) δ 8.34 – 8.24 (m, 2H), 8.12 (d, J = 2.5 Hz, 1H), 7.78 (s, 1H), 7.71 (s, 1H), 7.35 (d, J = 8.0 Hz, 1H), 7.10 (s, 1H), 6.79 (d, J = 9.3 Hz, 1H), 5.88 (s, 1H), 5.51 (d, 1H), 5.26 (s, 1H), 4.54 (d, 2H), 4.47 – 4.37 (m, 1H), 4.30 (t, 2H), 4.24 – 4.15 (m, 1H), 3.00 (t, 4H), 2.84 (dd, 1H), 2.65 (d, 1H), 2.20 (t, 2H), 2.03 (s, 2H), 1.52 – 1.42 (m, 3H), 1.28 (d, 3H). ^{13}C NMR (101 MHz, $CDCl_3$) δ 173.17, 154.50, 150.05, 137.01, 136.62, 127.45, 127.15, 123.89, 121.58, 121.18, 118.06, 109.65, 108.12, 66.49, 61.96, 59.95, 55.37, 40.42, 33.60, 29.95, 28.13, 24.55. MS (ESI-TOF) for $C_{29}H_{31}F_3N_6O_6S$ $[M + H]^+$ calculated 648.2051, found 648.2049.

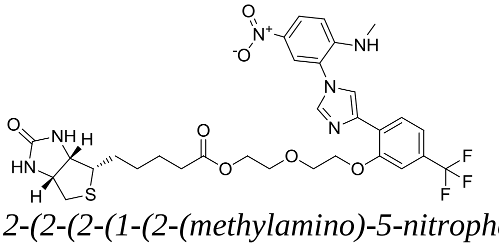

*2-(2-(2-(1-(2-(methylamino)-5-nitrophenyl)-1H-imidazol-4-yl)-5 (trifluoromethyl)phenoxy)ethoxy)ethyl 5-((3aS,4S,6aR)-2-oxohexahydro-1H-thieno[3,4-d]imidazol-4-yl)pentanoate (**47**).* Yellow solid (85 mg, 63 %). m.p. 53.2-54.7 °C.

^{1}H NMR (400 MHz, $CDCl_3$) δ 8.26 (dd, J = 9.2, 2.4 Hz, 1H), 8.17 (d, J = 7.9 Hz, 1H), 8.10 (d, J = 2.5 Hz, 1H), 7.88 (s, 1H), 7.73 (s, 1H), 7.32 (d, J = 8.2 Hz, 1H), 7.07 (s, 1H), 6.79 (d, J = 9.4 Hz, 1H), 6.14 (s, 1H), 6.02 (s, 1H), 5.76 (s, 1H), 4.49 (t, 2H), 4.24 m, 4H), 4.07 (s, 2H), 3.89 (s, 2H), 3.67 (m, 4H), 3.15 (s, 1H), 3.01 (d, J = 4.9 Hz, 3H), 2.88 (s, 1H), 2.84 (s, 1H), 2.17 (m, 2H), 1.66 (m, 2H), 1.53 (m, 4H). ^{13}C NMR (101 MHz, $CDCl_3$) δ 173.38, 164.72, 154.68, 150.17, 137.18, 136.44, 127.13, 125.47, 123.82, 121.84, 121.23, 117.80, 109.74, 108.16, 71.64, 68.86, 67.12, 63.08, 62.12, 60.32, 55.55, 40.61, 33.57, 31.52, 30.01, 28.37,

24.51, 22.58, 14.05, 2.93. MS (ESI-TOF) for $C_{31}H_{35}F_3N_6O_7S$ $[M + H]^+$ calculated 693.2313, found 693.2313.

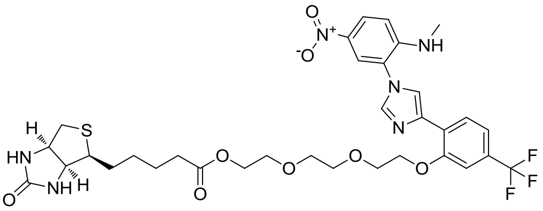

*2-(2-(2-(2-(1-(2-(methylamino)-5-nitrophenyl)-1H-imidazol-4-yl)-5-(trifluoromethyl)phenoxy)ethoxy)ethoxy)ethyl 5-((3aS,4S,6aR)-2-oxohexahydro-1H-thieno[3,4-d]imidazol-4-yl)pentanoate (**48**).* To a mixture of compound **23** (24.36mg, 0.05mmol) with anhydrous potassium carbonate (62.1 mg, 0.15 mmol) in acetone (8 mL). After 15 minutes the solution was added 2-(2-(2-iodoethoxy)ethoxy)ethyl 5-((3aS,4S,6aR)-2-oxohexahydro-1H-thieno[3,4-d]imidazol-4-yl)pentanoate (13.4 mg, 0.055 mmol). The mixture was reacted at 45 °C for 12 hours and was monitored by TLC. Then the crude mixture was stopped under concentrated pressure and extracted sequentially with ethyl acetate (3 × 50 mL). The organic layer was collected and dried over Na_2SO_4 and was again concentrated to dryness. The crude mixture was purified by column chromatography of alumina-B to afford the product as yellow solid (22.6 mg, 75%). m.p. 73.1-74.4 ° C. ^{1}H NMR (400 MHz, $CDCl_3$) δ 8.30 (dd, J = 9.1, 2.7 Hz, 2H), 8.15 (d, J = 2.6 Hz, 1H), 7.92 (d, J = 1.2 Hz, 1H), 7.76 (d, J = 0.9 Hz, 1H), 7.36 (d, J = 7.5 Hz, 1H), 7.17 (s, 1H), 6.78 (d, J = 9.3 Hz, 1H), 5.66 (s, 1H), 5.39 (d, 1H), 5.04 (s, 1H), 4.46 (dd, 1H), 4.34 – 4.27 (m, 2H), 4.25 (dd, 1H), 4.15 – 4.06 (m, 2H), 3.98 – 3.88 (m, 2H), 3.65 (dd, 2H), 3.51 (td, 4H), 3.10 (ddd, 1H), 2.98 (t, 3H), 2.88 (d, 1H), 2.71 (d, 1H), 2.28 (t, 2H), 1.68 – 1.53 (m, 4H), 1.45 – 1.30 (m, 2H). ^{13}C NMR (101 MHz, $CDCl_3$) δ 173.52, 163.75, 154.76, 150.12, 137.29, 136.60, 129.20, 127.05, 125.59, 123.79, 122.74, 121.74, 121.34, 117.74, 109.47, 108.20, 70.28, 69.36, 68.94, 67.27, 63.07, 61.83, 60.02, 55.48, 40.42, 33.58, 29.87, 28.21, 24.58. MS (ESI-TOF) for $C_{33}H_{39}F_3N_6O_8S$ $[M + H]^+$ calculated 737.2575, found 737.2576.
